# Assessment of light sensitivity using the Ocular Photosensitivity Analyzer: Discomfort threshold, pain threshold, and tolerance in healthy volunteers

**DOI:** 10.1371/journal.pone.0361637

**Published:** 2026-10-29

**Authors:** Nathan Goturi, Cameron Talbert, Nicholas Pondelis, Mariela C. Aguilar, Alex Gonzalez, Cornelis Rowaan, Heather Durkee, Jean-Marie Parel, Vanda Faria, Eric Alan Moulton

**Affiliations:** 1 Brain and Eye Pain Imaging Lab, Pain and Affective Neuroscience Center, Department of Anesthesia, Critical Care and Pain Medicine, Boston Children's Hospital and Harvard Medical School, Boston, Massachusetts, United States of America; 2 Ophthalmic Biophysics Center, Department of Ophthalmology, Bascom Palmer Eye Institute, University of Miami Miller School of Medicine, Miami, Florida, United States of America; 3 Department of Ophthalmology, Boston Children’s Hospital, Harvard Medical School, Boston, Massachusetts, United States of America; Harvard Medical School, UNITED STATES OF AMERICA

## Abstract

Photophobia encompasses a continuum of light-induced experiences from mild discomfort to overt pain and intolerance, yet clinical tools rarely quantify these distinctions, leaving many patients undiagnosed and untreated. This study employed the Ocular Photosensitivity Analyzer (OPA), a compact instrument for quantitative sensory testing (QST) with light, to investigate three perceptual levels in healthy adults: visual photosensitivity discomfort threshold (VPDT), visual photosensitivity pain threshold (VPPT), and an exploratory outcome, visual photosensitivity tolerance level (VPTol). Twenty-nine participants ages 19−34 underwent standardized OPA testing, during which they identified when light first became uncomfortable, painful, and intolerable. After each trial, participants rated the peak pain intensity (PI) and peak unpleasantness (UP) using visual analog scales. Measured illuminance values differed significantly across the perceptual levels (RM-ANOVA, N = 28, *F*(1.69, 45.6)=37.06, *p* < 0.001, *η*_*p*_*²* = 0.58; *Mauchly’s W* = 0.82, *p* = 0.072), indicating sensory levels are differentiable. Post hoc t-tests revealed VPTol exceeded VPPT (N = 28, *t*(27)=−2.29, *p* = 0.030, *d*_z_ = 0.43)), and bo*t*h exceeded VPDT (VPTol: N = 29, *t*(28)=−7.69, *p* < 0.001, *d*_z_ = 1.43, VPPT: N = 29, *t*(27)=−7.69, *p* < 0.001, *d*_z_ = 1.47). Friedman’s tes*t* revealed UP (N = 28, χ^2^(2)=17.64, *p* < 0.001, *Kendall’s W* = 0.32) and PI (N = 28, χ^2^(2)=22.36, *p* < 0.001, *Kendall’s W* = 0.40) differences across perceptual levels and post hoc Wilcoxon tests showed UP and PI increased between VPDT and VPPT (UP: N = 29, *Z* = −4.34, *p* < 0.001, *r =* 0.57, PI: N = 29, *Z* = −4.58, *p* < 0.001, *r =* 0.60) and UP increased between VPDT and VPTol (N = 28, *Z* = −2.92, *p* = 0.007, *r =* 0.39), suggesting that both affective and sensory dimensions of pain vary across perceptual levels. Although preliminary, these findings are consistent with a multidimensional framework of photophobia. Additionally, the OPA’s ability to differentially quantify VPDT, VPPT, and VPTol offers a novel approach to characterize light sensitivity that may aid future assessment of abnormal light sensitivity.

## Introduction

Photophobia is a disorder that may affect up to 25% of the population [1] and was originally described as exposure of the eye to light that induces or exacerbates pain [2]. It is a common feature in many ophthalmic and neurologic conditions, including migraine, traumatic brain injury, blepharospasm, and dry eye disease [3–7]. Since Lebensohn’s early work, our understanding of photophobia has evolved to a continuum of light-induced experiences ranging from mild discomfort to debilitating pain [5,6]. While photophobia remains intrinsically linked to the perception of pain, this broad definition complicates comparing light sensitivity within and between conditions, and standardizing labels of key perceptual experiences along this continuum is essential.

Perception of pain evoked by a noxious stimulus is a multidimensional experience that involves subjective evaluations of the sensory discriminative (intensity, duration, location) and affective (emotional) responses to the stimulus [8–11]. While these dimensions have been studied in various contexts, light-induced pain remains underexplored [12]. Furthermore, standard diagnostic techniques and treatments do not assess them, and many patients often leave clinic with no diagnosis or treatment for their light induced-pain [4], due in part to these characteristics being missed. To improve recognition and treatment, techniques are needed to assess photophobia’s multidimensional nature.

Quantitative sensory testing (QST) provides a method to interrogate pain mechanisms by assessing a person’s subjective reports (e.g., when they feel a stimulus first becomes painful) of sensory perceptions in response to calibrated stimuli [13]. QST has proven instrumental in uncovering pain mechanisms underlying neuropathic and chronic pain, such as central sensitization, by measuring sensory thresholds and suprathreshold responses [14–16], thereby advancing our understanding of somatosensory processing. Various groups have attempted to quantify light sensitivity [17–25]. Drawing standardized conclusions about photophobia has been difficult due to the differences in methodologies and parameters (light wavelength, stimulus duration, etc.) utilized. Quantitative studies require a standardized tool to measure photophobia in various populations [1].

One such tool was introduced by Verriotto et al. in 2017 with the Ocular Photosensitivity Analyzer (OPA), a compact automated instrument designed to present controlled light stimuli of varying intensities, enabling longitudinally reliable QST of visual photosensitivity [26]. Previous studies have demonstrated the OPA’s ability to identify visual photosensitivity discomfort thresholds (VPDT, often reported as “Visual Photosensitivity Threshold” or “VPT”: the level at which light becomes uncomfortable) [1,26–29]. Although many individuals experience other sensations of light sensitivity like pain and intolerance, it remains unclear if the OPA can differentially quantify visual photosensitivity pain thresholds (VPPT: the level at which light becomes painful) and visual photosensitivity tolerance levels (VPTol: the level at which light becomes intolerable). To bridge this knowledge gap, we assessed VPDT, VPPT, and VPTol with the OPA in healthy participants. Quantifying these perceptual levels is a first step towards establishing healthy reference ranges, enables future OPA studies in patients with various conditions, and advances integration of QST into investigations of light-induced pain, which may in turn support further efforts to refine assessment and inform future therapeutic research.

## Materials and methods

### Participants

The Boston Children’s Hospital (BCH) Institutional Review Board (IRB) approved all aspects of this research conducted (IRB-P00045728). The study met the guidelines for ethical human research set by the Declaration of Helsinki.

Healthy participants were recruited from the greater Boston metropolitan area from advertisement flyers and brochures placed at schools, libraries, and similar community-based centers, web advertisements such as the free community listserv ‘craigslist’, the “Find a Clinical Trial” section of BCH’s external website, an advertisement on the BCH internal website as a Research Announcement, and word-of-mouth.

Before consenting to the study, all potential participants were screened for inclusion and exclusion criteria. Initial screening was conducted via telephone, followed by an in-person interview to confirm eligibility. Participants were limited to young adults to minimize the effect of age-related ocular changes on light sensitivity measures. Eligible participants met inclusion criteria of: ≥ 18 years old, < 40 years old; no significant ocular surface pain (1-week recall average ocular pain intensity, Numeric Recall Score≤15, scale 0–100); no significant photophobia (1-week recall pain intensity, Numeric Recall Score≤15, scale 0–100); no significant dry eye symptoms (Ocular Surface Disease Index<13).

Exclusion criteria included: severe ocular conditions (corneal abnormalities [e.g., keratoconus, scarring], glaucoma, hard contact lens use, retinal abnormalities, scleritis, uveitis, history of corneal surgery); neurological disease (amyotrophic lateral sclerosis, Alzheimer’s, brain tumor, cerebrovascular accident, epilepsy or other seizure disorders, idiopathic hypersomnia, migraine, multiple sclerosis, neuropathy [e.g., chemotherapy-associated], Parkinson’s, severe traumatic brain injury, severe mental illness [DSM-5 axis I psychiatric disorder except anxiety and depression], spinal injury), inherited disorders associated with ocular manifestations (Bechet’s disease, Ehlers-Danlos syndrome; Fabry disease); medical conditions with potential ocular manifestations (diabetes; Lupus; HIV; sarcoidosis; thyroid disease). These criteria were evaluated based on self-report during pre-screening; participants did not undergo examinations or testing for study purposes. Participants that met the criteria were considered healthy for the purposes of this study. All participants provided written informed consent before any study procedures began.

### QST protocol

QST was performed with the OPA [1,26] (Bascom Palmer Eye Institute, Miami, FL) (Fig 1). All participants received consistent scripted verbal and computer instructions (S2 File). Testing was binocular and conducted in a dark room to minimize effects of light not produced by the OPA. Pupil size was not monitored.

**Fig 1:**
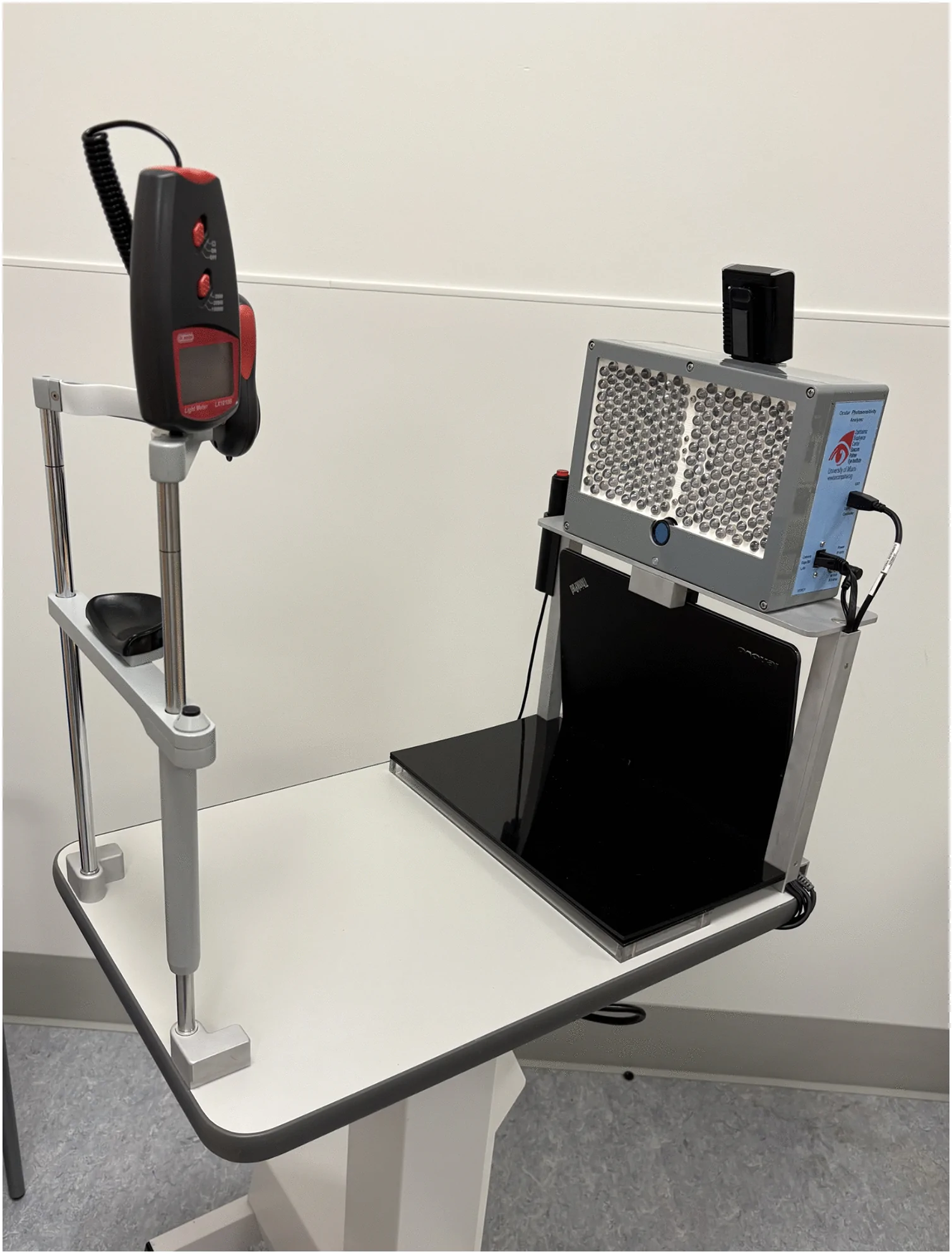
The Ocular Photosensitivity Analyzer. Displays a side-view of the head-chin rest, lux meter, and LED array panel.

Participants’ faces were positioned 50 cm from the light source consisting of 210 white light emitting diodes (LED) (COM-11118, White – 10 mm, Sparkfun, Niwot, CO) and they were given the OPA’s handheld push-button [1]. The LED array and handheld push-button (Delcom Engineering, Rye Brook, NY) were controlled using a touch screen laptop computer (Lenovo ThinkPad Twist S230u, Lenovo, Morrisville, NC) operating a customized control application developed with LabVIEW System Design Software (National Instruments, Austin, TX) [1]. The OPA produces light stimuli of varying intensities (range: 0–4.5 log lux), starting with the dimmest light stimulus and gradually increasing intensity, utilizing unequal ascending and descending steps according to the Garcia-Perez staircase technique [1]. Each light stimulus is presented for 2 seconds, followed by a rest period of 4 seconds.

To evaluate VPDT, participants were instructed to indicate whether the light stimulus was “uncomfortable” by pressing the hand-held button. Each threshold was calculated from the mean of 10 response reversals. A higher threshold indicates less sensitivity to light while a lower threshold indicates greater sensitivity to light. The same protocol and calculation process was used to evaluate VPPT, in which participants were directed to press the handheld button when the light stimulus first becomes “painful”. To evaluate VPTol, rather than the OPA increasing light intensity in discrete steps in an automated manner, instead light intensity was manually increased by the operator in a gradual manner such that it would require approximately 30 seconds to reach the OPA’s maximum output of 4.5 log lux. Participants were instructed to tell the operator to stop when they could no longer tolerate the light, without squinting. Squinting was not monitored and the test did not stop if a participant squinted involuntary. The light intensity at that point was measured with a digital lux meter (LX1010B, Dr. meter, Thousandshores Inc, Union City, CA) to determine VPTol. The same operator conducted all VPTol evaluations to ensure consistency in the manual light intensity ramp. This test was performed once per participant.

Participants completed three trials wearing clear lenses in the following order of evaluations: VPDT, then VPPT, then VPTol (Fig 2). Clear lenses were plano lenses and standardized across participants. They were chosen to minimize lens effects on the perceptual thresholds measured, while providing a baseline for comparison to make this data comparable with other studies that use lens tints. The order of evaluations was chosen to progress from the least to the most intense stimulus, thereby reducing the amount of anticipated photoreceptor bleaching with higher intensity light prior to each trial. Each trial was preceded by a 10-minute dark adaptation period. Questionnaires, including a demographics, current medications, and comorbidities inventory, were administered verbally during the dark adaptation periods.

**Fig 2:**
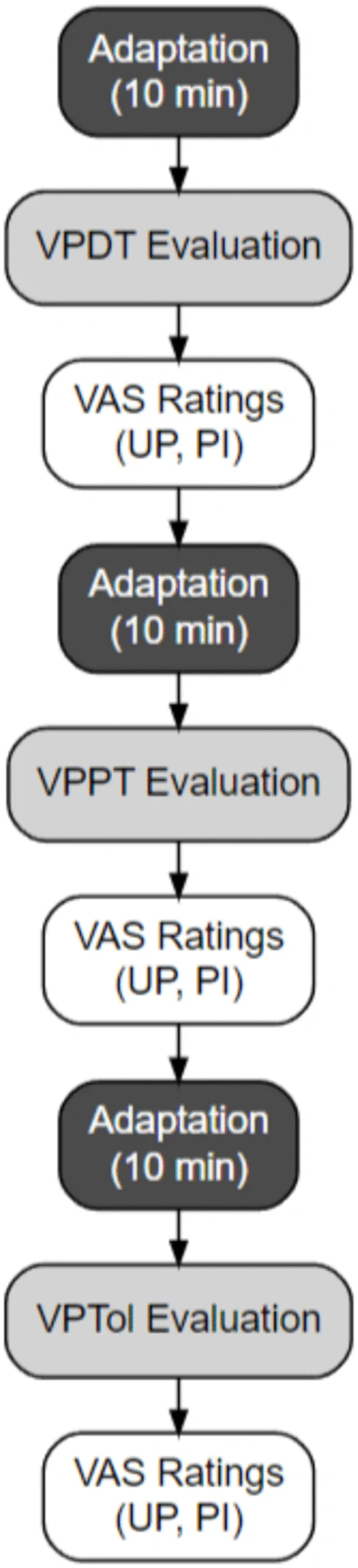
QST Battery. Participants completed three QST trials in the order: VPDT, VPPT, and VPTol. Prior to each trial, there was a 10-minute adaptation period. After each trial, participants rated unpleasantness (UP) and pain intensity (PI) on VAS.

After the first trial evaluating VPDT, participants were read a statement differentiating between pain and unpleasantness: “Pain intensity is how strong the pain sensation is, while unpleasantness is how much it bothers you. They are related, but different. Consider the example of having a knife lightly resting on the back of your neck – though it may not be painful, it would likely be unpleasant”. Participants were asked if they remembered the difference between pain and unpleasantness after the second trial evaluating VPPT, and were reminded of the difference if they could not remember it.

After each trial and any relevant clarification of pain versus unpleasantness, participants were presented with two computerized Visual Analog Scales (VAS). The first VAS was labeled Pain Intensity with “no pain at all” on the left side of the scale and “the most intense pain imaginable” on the right side. The second VAS was labeled Unpleasantness with “not unpleasant at all” on the left side and “the most unpleasant sensation imaginable” on the right side. Participants were told to adjust the computerized sliders on each VAS to rate the peak pain intensity (PI) and peak unpleasantness (UP) they felt during each OPA trial. VAS ratings of PI and UP of the sensations were scaled to 0–100 for quantification, with higher numbers indicating more severe pain intensity or greater unpleasantness, respectively.

Study data were collected and managed using REDCap (Research Electronic Data Capture), a secure, web-based software platform designed to support data capture for research studies, hosted at BCH [30,31].

### Data analysis

Data normality was assessed using Shapiro-Wilk tests. Descriptive statistics are reported as Mean ± Standard Deviation for normally distributed variables or Median with Interquartile Range (IQR) for non-normally distributed variables. The choice of parametric or non-parametric statistical approach was guided directly by the outcome of the Shapiro-Wilk test for each variable (S2 Table).

Shapiro-Wilk testing indicated the measured illuminance values for all perceptual levels were normally distributed and thus statistical analyses conducted on this data were parametric. As such, a repeated measures analysis of variance (RM-ANOVA) was conducted to assess differences in measured illuminance across the perceptual levels: VPDT, VPPT, and VPTol. Sphericity was assessed for all within-subjects effects, and Greenhouse-Geisser-corrected degrees of freedom (ε = 0.85) are reported as a conservative measure. A mixed-design RM-ANOVA with the within-subjects factor of perceptual level (VPDT, VPPT, and VPTol) and the between-subjects factor of sex assigned at birth (male vs. female) was conducted to examine the effect of sex. Post-hoc paired t-tests were performed to evaluate the relative differences in illuminance to evoke VPDT, VPPT, and VPTol, with p-values manually adjusted for multiple comparisons using the Holm correction.

Shapiro-Wilk testing indicated the UP and PI VAS ratings for all perceptual levels were generally non-normally distributed and thus statistical analyses conducted on this data were non-parametric. Similarly, descriptive statistics are reported as median (IQR). A Friedman’s test was conducted to assess differences in UP ratings across the three perceptual levels: VPDT, VPPT, and VPTol. A separate Friedman’s test was conducted to assess differences in PI ratings across these perceptual levels. Mann-Whitney U tests were performed separately at each perceptual level (VPDT, VPPT, VPTol) to test the effect of sex by comparing participants assigned male and female at birth. Wilcoxon signed-rank tests were employed for post-hoc comparisons of UP and PI ratings within and across these testing conditions to assess subjective experience, with p-values manually adjusted for multiple comparisons using the Holm correction.

A p-value less than 0.05 was considered statistically significant. Statistical analyses were performed using SPSS version 29.0 software (IBM, Armonk, NY).

## Results

### Participant demographics

A total of 31 individuals were recruited. Two subjects were not able to complete full OPA assessment due to technical malfunctions with the device. Twenty-nine healthy adult participants ages 19–34 years old, with a mean age of 24.0 ± 4.1 years old completed full OPA assessment. One participant reached the OPA’s maximum output during the VPPT test and their lux value was excluded analytically, though the UP and PI values were included. A separate participant completed VPTol assessment, but did not record UP or PI values, and thus only their lux value was included in analysis. Fifty two percent of participants were assigned female at birth. Comorbidities and medications are presented in S3 Table.

### Discomfort threshold, pain threshold, and tolerance to light are distinguishable

The illuminance measurements in log lux for VPDT, VPPT, and VPTol were 1.7 ± 0.8, 2.5 ± 1.1, and 2.9 ± 1.1, respectively (Fig 3). An RM-ANOVA revealed significant differences in measured illuminance for the three tested perceptual levels (N = 28, *F*(1.69, 45.6))=37.06, *p* < 0.001, *η*_*p*_*²* = 0.58; *Mauchly’s W* = 0.82, *p* = 0.072), indicating QST using the OPA successfully differentiated between VPDT, VPPT, and in an exploratory manner, VPTol in healthy participants. Post hoc comparisons showed that VPPT values were significantly greater than those for VPDT (N = 28, mean difference = 0.82, 95% CI [0.60,1.03], *t*(27)=−7.69, *p_adj* < 0.001, *d*_z_ = 1.47) and *t*hough not a direct comparison, VPTol values were significantly greater than both those for VPPT (N = 28, mean difference = 0.35, 95% CI [0.04,0.66], *t*(27)=−2.29, *p_adj* = 0.030, *d*_z_ = 0.43) and VPDT (N = 29, mean difference = 1.15, 95% CI [0.84,1.45], *t*(28)=−7.69, *p_adj* < 0.001, *d*_z_ = 1.43) based on *t*wo-*t*ailed paired samples t-tests.

**Fig 3:**
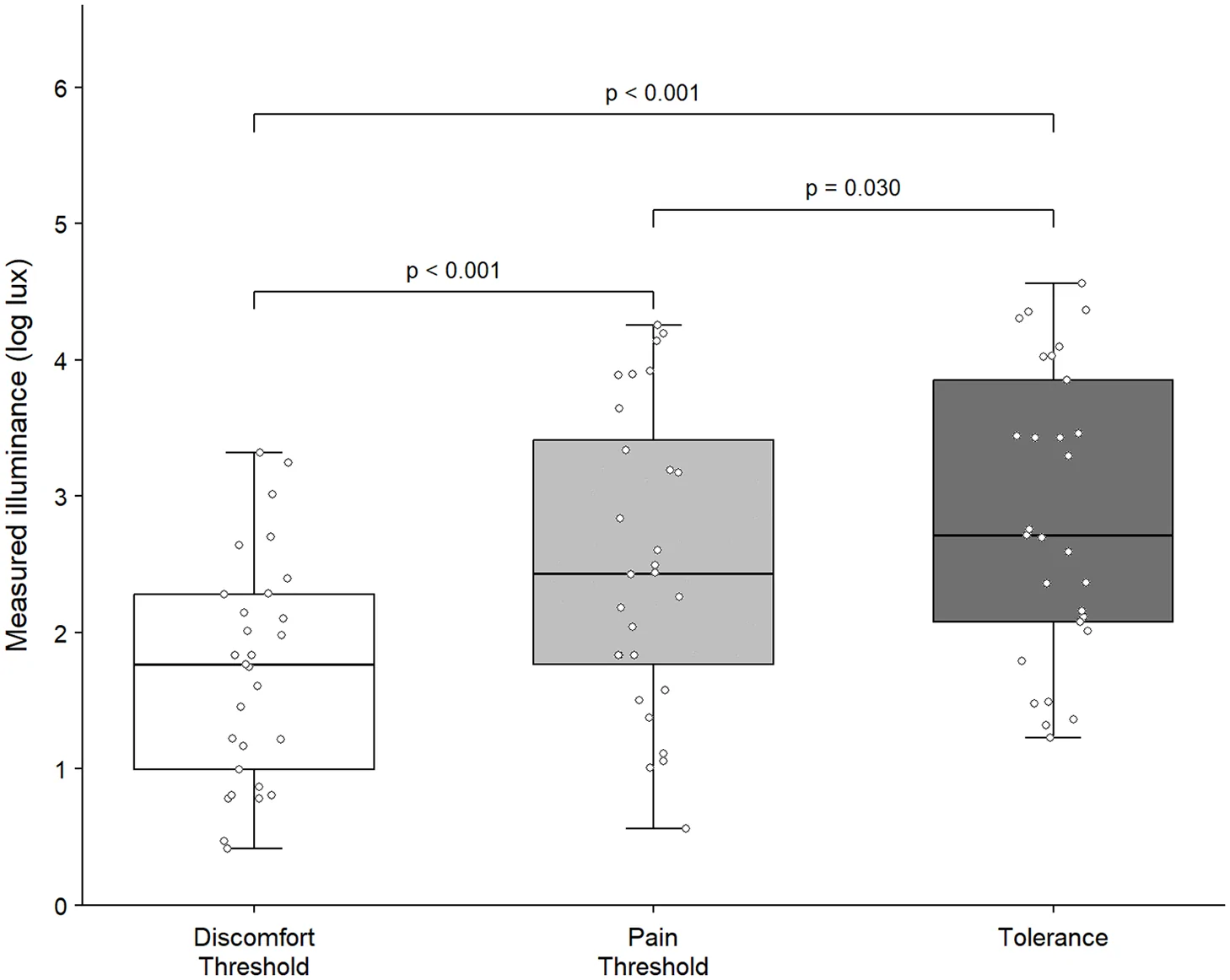
Comparison of Perceptual Thresholds. Box-and-whisker plots for measured illuminance in VPDT, VPPT, and VPTol testing across 29 healthy adult participants. Illuminance values in log lux were significantly different between all perceptual levels with tolerance exceeding pain threshold and both exceeding discomfort threshold. Individual participant ratings are plotted as dots.

A mixed design RM-ANOVA with the within-subjects factor of perceptual level and the between-subjects factor of sex assigned at birth revealed that there were no significant main effect of sex (N = 28, *F*(1, 26)=1.00, *p* = 0.326, *η*_*p*_*²* = 0.04) and no sex * perceptual level interaction (N = 28, *F*(1.70, 44.1)=0.34, *p* = 0.676, *η*_*p*_*²* = 0.01).

### Pain intensity and unpleasantness ratings at each perceptual level

UP ratings for VPDT, VPPT, and VPTol testing were 25.0 (IQR = 18.0–48.0), 59.0 (IQR = 36.0–75.0), and54 (IQR = 34.8–66.3), respectively. PI ratings for VPDT, VPPT, and VPTol were 5.0 (IQR = 0–14.0), 19.0 (IQR = 9.0–28.0), and 14.5 (IQR = 0–21.8), respectively. All UP and PI ratings are reported as median and IQR in Fig 4.

**Fig 4:**
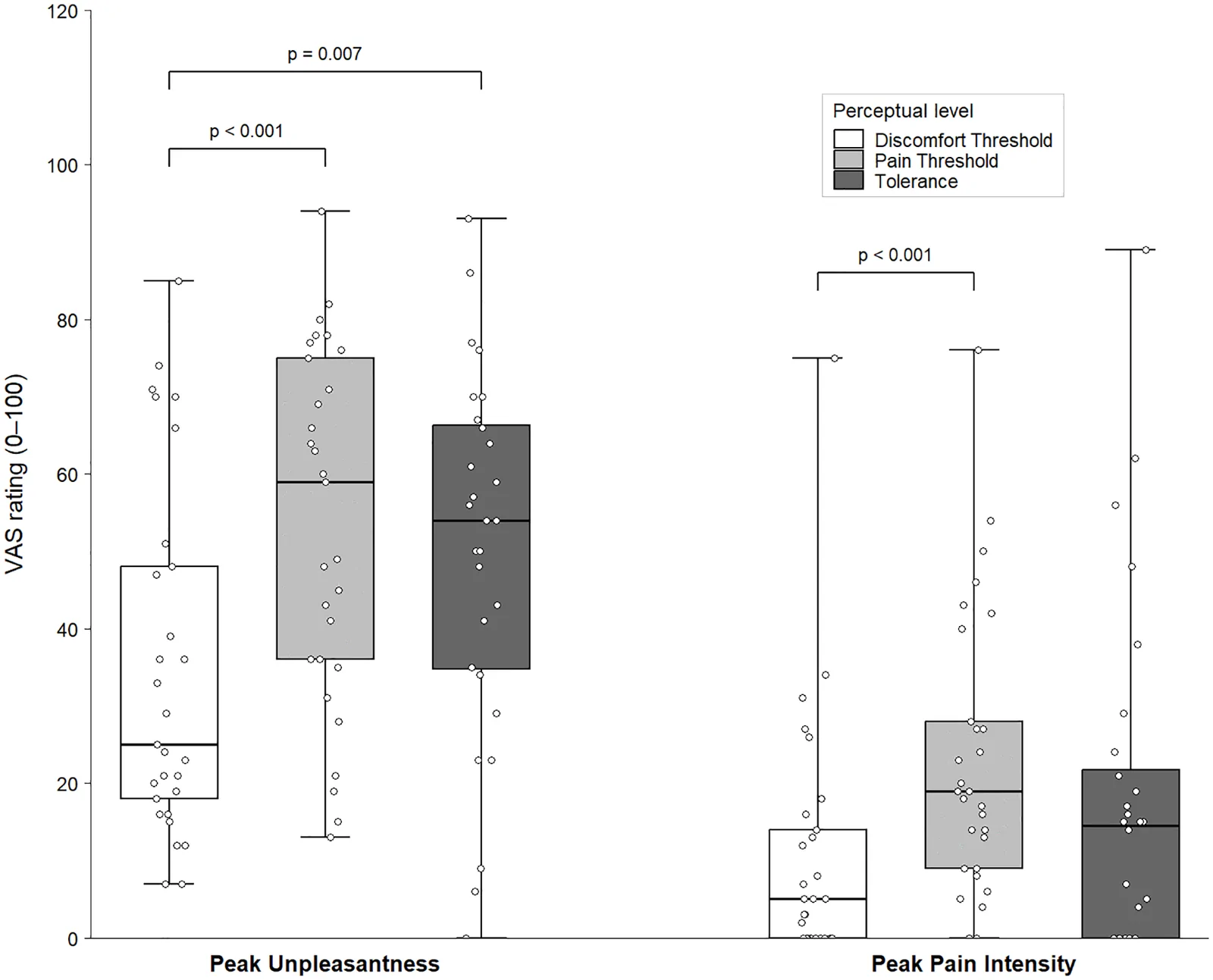
UP and VAS Ratings. Median UP and PI VAS ratings for VPDT, VPPT, and VPTol testing. VAS ratings were significantly different such that those for pain threshold exceeded discomfort threshold. Furthermore, UP ratings exceeded PI ratings at all perceptual levels. Individual participant ratings are plotted as dots.

Friedman’s tests revealed significant differences in UP (N = 28, *χ*^*2*^(2)=17.64, *p* < 0.001, *Kendall’s W* = 0.32) and PI (N = 28, *χ*^*2*^(2)=22.36, *p* < 0.001, *Kendall’s W* = 0.40) ratings across the three tested perceptual levels. For each testing condition, UP ratings were significantly greater than PI ratings at each perceptual level: VPDT (N = 29, *Z* = 4.62, *p_adj* < 0.001, *r =* 0.61), VPPT (N = 29, *Z* = 4.60, *p_adj* < 0.001, *r =* 0.60) and VPTol (N = 28, *Z* = 4.54, *_adj p* < 0.001, *r =* 0.61). VPPT testing resulted in significantly higher UP (N = 29, *Z* = −4.34, *p_adj* < 0.001, *r =* 0.57) and PI (N = 29, *Z* = −4.58, *p_adj* < 0.001, *r =* 0.60) ratings compared to VPDT testing. VPTol testing also produced significantly higher UP (N = 28, *Z* = −2.92, *p_adj* = 0.007, *r =* 0.39) ratings compared to VPDT testing. However, VPTol testing did not result in significantly greater PI ratings (N = 28, *Z* = −2.14, *p_adj* = 0.065, *r =* 0.29) compared to VPDT testing. Furthermore, VPTol testing did not yield greater UP (N = 28, *Z* = −1.06, *p_adj* = 0.289, *r =* 0.14) or PI (N = 28, *Z* = −1.87, *p_adj* = 0.065, *r =* 0.25) ratings as compared to VPPT testing. Mann-Whitney U testing revealed there were no sex differences across UP and PI ratings at each perceptual level (S4 Table).

## Discussion

In this study, the OPA successfully differentiated three perceptual levels of light sensitivity in healthy volunteers: discomfort (VPDT), pain (VPPT), and tolerance (VPTol). While previous studies have shown the OPA can assess VPDT (sometimes reported as “Visual Photosensitivity Threshold” or “VPT”) [1,26–29], this work is the first to demonstrate the OPA’s ability quantify and differentiate VPPT and VPTol. Though comparisons with VPTol are limited by the methodological differences from VPDT and VPPT testing, participants exhibited a graduating trend in the respective measured illuminance values such that discomfort was less than pain and both were less than tolerance. While we did not formally assess whether participants interpreted the constructs of pain, discomfort, and tolerance uniformly, our work provides initial evidence supporting these conceptual distinctions by showing that subjects can distinguish them with instructions.

### OPA can measure distinct perceptual levels along a range of light sensitivity

The separably quantifiable levels of discomfort threshold, pain threshold, and tolerance indicate that each may represent a distinct sensory experience along the perceptual continuum of light sensitivity. The progression in measured illuminance across the tested perceptual levels provides preliminary evidence that light-induced pain may not be a unitary phenomenon as originally described [2], but rather exists along this perceptual continuum. A perceptual continuum of light sensitivity is better aligned with the variable nature of photophobic presentations, with chief complaints ranging from headache to dry eyes to diplopia [4].

The ability of the OPA to differentiate these perceptual levels highlights its utility as a tool for QST of visual photosensitivity. Though the perceptual levels were operationally defined by task instructions, the within-subjects paradigm only required that each participants apply the instructions consistently, without assuming shared semantic interpretations across subjects. Thus, by providing discrete VPDT, VPPT, and VPTol values, the OPA offers an index of various points at which an individual may feel affected by light. Additionally, we are not aware of any prior attempts to quantify VPTol. While it remains exploratory due to the operator-controlled ramp-up testing differing from the algorithm-controlled assessments of VPDT and VPPT, its inclusion extends the potential range of measurable responses and offers new insight into the wide range of light-induced pain. This multi-level characterization of the light pain response is a novel contribution. By enabling characterization of different levels of light sensitivity, the OPA may facilitate future studies into underlying mechanisms that differentiate these perceptual experiences and offer improved characterization of symptoms.

A multidimensional framework of pain [11] aligns with the concept that it may manifest at multiple perceptual levels including discomfort threshold, pain threshold, and tolerance. Differing from traditional QST paradigms classically focused on stimulus detection, pain detection, and tolerance, discomfort threshold was the criteria of OPA testing from its inception to better capture the amorphous and abstract nature of photophobia [1,26]. By capturing and differentiating additional perceptual levels, our work extends prior characterization of light-induced discomfort and pain. The few studies assessing discomfort threshold in alternate QST modalities consistently demonstrate that pain threshold exceeds discomfort threshold [32–35] but is exceeded by pain tolerance [36–38]. Our findings that VPPT exceeded VPDT and VPTol exceeded VPPT suggest a similar hierarchy may exist in light-induced pain.

### Subjective ratings suggest that light-induced pain demonstrates greater UP than PI

Our results suggest that both the sensory and affective dimensions contribute to light-induced pain sensation [11]. Previous studies have shown that these dimensions are intrinsically related, with different perceptual levels representing various levels of contribution from two to comprise the respective sensation of pain [10,39]. While UP and PI ratings are not direct correlates of these affective and sensory dimensions of pain, they serve as approximations in experimental pain research [40,41]. In this study, participants rated UP higher than PI at all perceptual levels. Higher UP ratings relative to those for PI align with previous findings in QST studies, particularly for stimuli with a strong anticipatory or affective component [41–43]. The brief pauses between the increasing discrete light intensities may have resulted in this anticipatory effect in VPDT and VPPT testing, and the pre-test instructions for the VPTol test may have done the same. Alternatively, light-induced pain may inherently engage stronger affective processing. Light stimuli, especially in sensitive individuals, can evoke immediate limbic responses that enhance unpleasantness, independent of the nociceptive intensity of the stimulus itself [5,44]. Furthermore, instructions and contextual framing of rating scales are known to affect how individuals perceive and report pain [12], and thus the explicit definitions provided for PI and UP may have influenced participants’ subjective evaluations.

PI ratings plateaued between VPDT and VPTol and both UP and PI ratings plateaued between VPPT and VPTol, even though measured illuminance for VPTol exceeded VPPT and VPDT. While it is possible participants reached a maximum rating beyond which no further increases in subjective experience are reported despite escalating stimulus intensities, the moderate UP and low PI ratings indicate this was unlikely. The duration of the stimulus intensity at VPTol was quite brief and ended upon detection in contrast to VPDT and VPPT. Further, the gradual ramp of light in the VPTol test may have resulted in a temporal summation effect that caused the illuminance to feel more intolerable than it would have if presented discretely. The manual adjustment of light intensity during VPTol measurements introduced potential variability, which may have contributed to the lack of differentiation between VPPT and VPTol ratings. Finally, all VAS ratings were retrospective and the timing difference between testing and rating may have also contributed to subjective evaluations, emphasizing the importance of refining future protocols to minimize ambiguity.

Our findings reinforce the importance of considering both sensory and affective dimensions when evaluating light-induced pain responses. Doing so may allow for a more comprehensive understanding of photophobia, which may ultimately inform the development of more precise diagnostic tools and targeted interventions for patients experiencing light sensitivity.

### Clarifying “photophobia”: photodysesthesia vs. photoallodynia

Though photophobia is known to exist along a continuum [5,6], our work provides preliminary evidence suggesting the existence of key points – discomfort, pain, and tolerance – along the range of light sensitivity that can be differentially activated in healthy subjects. Patients experience light sensitivity along a continuum [44], however, they are all labeled with photophobia, an umbrella term covering any light-triggered symptom (ocular or central) leading to light sensitivity. Under this vague label, two patients could have vastly different experiences – one feels slight burning in the eyes in sunlight, while another gets stabbing ocular pain even in moderate light – yet both are labeled equally as “photophobic”. This oversimplification hinders our ability to tailor treatments. To address the gap between available terminology and these sensory experiences we propose, for consideration and further evaluation, more precise terminology: “photodysesthesia” to denote sensations of abnormal discomfort induced by light, and “photoallodynia” to describe abnormal pain sensations triggered by light exposure. Labelling light-induced pain during a normal eye exam as photoallodynia in a study of corneal neuropathy allowed for its isolation and subsequent treatment, confirming the term’s utility [45] and suggesting that greater specification may lead to improved diagnosis and clinical outcomes. However, not all photophobia is overly painful and thus we offer photodysesthesia, adopting -dysesthesia for an unpleasant abnormal sensation. Since patients with photodysesthesia might have different etiologies than those with photoallodynia, distinguishing between the two may facilitate improved understanding, diagnosis, and treatment of conditions involving abnormal light sensitivity. Further work is needed to clarify these proposed terms and validate their utility.

### Limitations

Limitations of this study include the use of only young volunteers whose conditions were self-reported and no formal ocular examinations were performed. Thus they may have exhibited different responses than different age groups and clinical populations experiencing abnormal light sensitivity. Additionally, not all participants completed the full testing protocol, potentially affecting the representativeness of the data. We did not perform formal validation of the discomfort, pain, tolerance, unpleasantness, and pain intensity constructs, nor did we assess test-retest reliability. These categories are inherently subjective and the test instructions may have been interpreted differently by participants. Furthermore, the instructions explicitly defining pain versus unpleasantness are subject to the same variability in semantic understanding and may have also contributed to the observed differentiations of perceptual levels and UP and PI. Though Holm corrections were applied, the relatively small cohort (N = 29) combined with the number of post-hoc comparisons increases the risk of unstable estimates. The fixed testing order was not counterbalanced, confounding sequence with perceptual level. This may have also introduced anticipation, habituation, sensitization, anchoring, learning, or carryover effects during assessments. Finally, manual adjustment during VPTol testing introduced further confounders including temporal summation, adaptation, hysteresis, operator variability, expectancy, and inconsistent stimulus kinetics. Additionally, squinting was not monitored and the VPTol test did not stop if a participant squinted involuntary. Thus our VPTol findings and comparisons remain exploratory and must be interpreted with caution. Future research should investigate these perceptual levels in large samples of healthy individuals of all ages and patient populations with disorders characterized by abnormal light sensitivity, establish test-retest reliability and construct validity for all terms subject to variation in semantic understanding, and use automated VPTol testing.

## Conclusions

The OPA’s ability to quantify distinct perceptual thresholds represents a methodological advance in the assessment of light-induced pain. It has the potential to be a clinically suitable tool for QST of light sensitivity [1] and further work should investigate its utility in this clinical setting. Whereas further investigation is needed to establish normative reference ranges for these perceptual levels in healthy participants, the OPA may enhance clinical trials by allowing for the evaluation of various sensory responses, including photodysesthesia and photoallodynia across various ophthalmic and neurologic pathologies. Future work should evaluate the relationship between light and anticipatory pain processing. Additionally, combining OPA stimuli with neuroimaging could elucidate the dynamic activation of involved neural pathways. Adopting more precise terminology and leveraging tools like the OPA may eventually improve clinical diagnosis, guide personalized treatments, and facilitate research into underlying pathophysiological mechanisms. Ultimately, these advancements may improve quality of life for patients experiencing abnormal light sensitivity.

## Supporting information

S1 File Analysis Syntax. (DOCX)

S1 Data Data formatted for analysis. (XLSX)

S2 File QST Protocol Full Script. (DOCX)

S2 Table Shapiro-Wilk test for normality for all outcome variables. One participant completed full OPA assessment but did not enter VAS ratings for UP and PI in the Tol test. A separate participant had no VPPT measured illuminance value due to technical error recording it, but otherwise completed full OPA assessment. This participant’s VAS ratings for the VPPT test were present and included in the analysis.
(DOCX)

S3 Table Comorbidities and medications. Displays comorbidities and medications for study participants. Medications taken by participants were stable and taken for ≥4 weeks. While not linked with alteration of pain perception, starting or stopping some of the included medications (e.g., systemic birth control) can affect mood or inflammatory background, and so in turn affect psychometric data.
(DOCX)

S4 Table Sex differences in measured illuminance values. Mann-Whitney U tests to assess for sex differences in measured illuminance values, UP ratings, and PI ratings for VPDT, VPPT, and VPTol.
(DOCX)
