# Supplementary material for: Assessment of light sensitivity using the Ocular Photosensitivity Analyzer: Discomfort threshold, pain threshold, and tolerance in healthy volunteers
Source: PLoS One. 2026 Oct 29;21(10):e0361637. doi: 10.1371/journal.pone.0361637 (PMC13718949; doi:10.1371/journal.pone.0361637)
Supplement: Supplementary file 1 [file pone.0361637.s001.docx]

Analysis Syntax – SPSS

**1. Tests of normality**

EXAMINE VARIABLES=VPDT VPPT VPTol VPDT_UP VPPT_UP VPTol_UP VPDT_PI VPPT_PI VPTol_PI

/PLOT BOXPLOT STEMLEAF NPPLOT

/COMPARE GROUPS

/STATISTICS DESCRIPTIVES

/CINTERVAL 95

/MISSING PAIRWISE

/NOTOTAL.

**2. RM-ANOVA to assess differences in measured illuminance across VPDT, VPPT, and VPTol**

GLM VPDT VPPT VPTol

/WSFACTOR=level 3 Polynomial

/METHOD=SSTYPE(3)

/EMMEANS=TABLES(level) COMPARE ADJ(LSD)

/PRINT=DESCRIPTIVE ETASQ

/CRITERIA=ALPHA(.05)

/WSDESIGN=level.

**3. Mixed-design RM-ANOVA with the within-subjects factor of perceptual level (VPDT, VPPT, and VPTol) and the between-subjects factor of sex assigned at birth (male vs. female)**

GLM VPDT VPPT VPTol BY Sex

/WSFACTOR=level 3 Polynomial

/METHOD=SSTYPE(3)

/CRITERIA=ALPHA(.05)

/WSDESIGN=level

/DESIGN=Sex.

**4. Post-hoc t-tests for perceptual levels** (*Holm-corrected p-values were calculated manually)

T-TEST PAIRS=VPPT VPTol VPTol WITH VPDT VPDT VPPT (PAIRED)

/CRITERIA=CI(.95)

/MISSING=ANALYSIS.

**5. Friedman’s test (UP)**

NPAR TESTS

/FRIEDMAN=VPDT_UP VPPT_UP VPTol_UP

/KENDALL=VPDT_UP VPPT_UP VPTol_UP

/STATISTICS DESCRIPTIVES QUARTILES

/MISSING LISTWISE.

**6. Friedman’s test (PI)**

NPAR TESTS

/FRIEDMAN=VPDT_PI VPPT_PI VPTol_PI

/KENDALL=VPDT_PI VPPT_PI VPTol_PI

/STATISTICS DESCRIPTIVES QUARTILES

/MISSING LISTWISE.

**7. Mann-whitney U tests for UP and PI**
NPAR TESTS

/M-W=VPDT_UP VPPT_UP VPTol_UP VPDT_PI VPPT_PI VPTol_PI BY SexN(1 2)

/MISSING ANALYSIS.

**8. Wilcoxon comparisons UP vs. PI within each perceptual level**

NPAR TESTS

/WILCOXON=VPDT_UP VPPT_UP VPTol_UP WITH VPDT_PI VPPT_PI VPTol_PI (PAIRED)

/MISSING ANALYSIS.

**9. Wilcoxon comparisons UP only – across perceptual levels**

NPAR TESTS

/WILCOXON=VPPT_UP VPTol_UP VPTol_UP WITH VPDT_UP VPDT_UP VPPT_UP (PAIRED)

/MISSING ANALYSIS.

**10. Wilcoxon comparisons PI only – across perceptual levels**

NPAR TESTS

/WILCOXON=VPPT_PI VPTol_PI VPTol_PI WITH VPDT_PI VPDT_PI VPPT_PI (PAIRED)

/MISSING ANALYSIS.
