# Supplementary material for: Assessment of light sensitivity using the Ocular Photosensitivity Analyzer: Discomfort threshold, pain threshold, and tolerance in healthy volunteers
Source: PLoS One. 2026 Oct 29;21(10):e0361637. doi: 10.1371/journal.pone.0361637 (PMC13718949; doi:10.1371/journal.pone.0361637)
Supplement: Supplementary file 2 [file pone.0361637.s003.docx]

**Supplementary Protocol: QST Protocol Full Script**

The data analyzed in this study were part of a larger protocol designed to address additional questions beyond the scope of this manuscript. The relevant sections from the protocol script have been included in this supplementary methods section.

**Test 1 – VPDT Assessment**

Technician: “The goal of this study is to investigate light sensitivity using the Ocular Photosensitivity Analyzer. The study’s purpose is to evaluate visual light sensitivity thresholds and determine whether light sensitivity is changed with different testing instructions in healthy individuals. You will complete questionnaires about eye symptoms and undergo a series of visual photosensitivity tests. This study will take approximately 1.5 hours. The total study compensation for your time and travel is $75.00. If you do not complete the study, you will be compensated for the testing you do complete. Your participation is voluntary. You can decline to participate, and you can stop your participation at any time, if you wish to do so, without any negative consequences to you. While this is not a treatment study, the results can help individuals with visual photosensitivity in the future.  Do you have any questions?”

Technician:  “For today’s testing we will use this device to determine how sensitive your eyes are to light. During this test, you will be wearing a lens designated as a placebo or fake lens - which means a control lens lacking the active filtering properties of the test lenses. Placebo lenses do not contain any specialized filters and are not expected to reduce light sensitivity or other symptoms during the testing period. Using placebo lenses is a standard and essential procedure in clinical research aimed at rigorously evaluating the true effectiveness of the active lens. Please remember not to squint or close your eyes during this test and you will hear the audio instructions in a moment.

OPA: “Hi, my name is IRIS. In a few moments, I will perform several measurements to determine how sensitive your eyes are to light. I will be turning the light on and off at different times, and also changing the intensity of the light. It is important that at all times during this test you look straight ahead and focus on the white blinking point in front of you. After each measurement, I will turn off the light and I will ask you to press the button in your hand only if you felt that the light intensity was uncomfortable. This pattern of the light turning on and off and you indicating whether it was uncomfortable will repeat multiple times. I will tell you when the test is finished.

Let me repeat the important points once again.

1. Always look straight ahead at the blinking point at all times during the entire test whether the light is on or off.

2. If the light intensity feels uncomfortable, wait for it to turn off and then press the button.

3. If the light intensity does not feel uncomfortable, do not press the button.

Great, do you have any questions? If not, please press the button to begin the test.”

*OPA conducts VPDT assessment.*

Technician: “Please consider how each light instance made you feel. Think about the sensations you experienced in terms of two separate dimensions of pain: pain intensity and unpleasantness. Pain intensity is how strong the pain sensation is, while unpleasantness is how much it bothers you. They are related, but different. Consider the example of having a knife lightly resting on the back of your neck – though it may not be painful, it would likely be unpleasant. Please rate the maximum pain intensity you just experienced with light using this sliding scale, with markings from none to the most imaginable. Now rate the maximum unpleasantness.”

*Participants rates UP and PI for VPDT test.*

Technician: “Excellent, that concludes this first test, we will have a short rest period before we complete the next test.”

**Test 2 – VPPT Assessment**

Technician: This test is computer guided and will operate similarly to the first test. You will be wearing the same placebo lenses for this test. However, this time around, you will only press the button if you feel pain. Please remember not to squint or close your eyes during this test and you will hear the audio instructions in a moment.

OPA: “Hi, my name is IRIS. In a few moments, I will perform several measurements to determine how sensitive your eyes are to light. I will be turning the light on and off at different times, and also changing the intensity of the light. It is important that at all times during this test you look straight ahead and focus on the white blinking point in front of you. After each measurement, I will turn off the light and I will ask you to press the button in your hand only if you felt that the light intensity was painful. This pattern of the light turning on and off and you indicating whether it was painful will repeat multiple times. I will tell you when the test is finished.

Let me repeat the important points once again.

1. Always look straight ahead at the blinking point at all times during the entire test whether the light is on or off.

2. If the light intensity feels painful, wait for it to turn off and then press the button.

3. If the light intensity does not feel painful, do not press the button.

Great, do you have any questions? If not, please press the button to begin the test.”

*OPA conducts VPPT assessment.*

Technician: “Remember the difference between pain intensity and unpleasantness?”

* Ask patient to repeat, and help them if they don’t remember.*

“Please rate the maximum pain intensity you just experienced with light using this sliding scale, with markings from none to the most imaginable. Now rate the maximum unpleasantness.”

*Participant rates UP and PI for VPPT test.*

Technician: “Excellent, that concludes this second test. We will have a short rest period before we complete the next test.

**Test 3 – VPTol Assessment**

Technician: “Now, we want to see how much light you can tolerate. This next test is manually operated. First, I will be turning the light on, and then gradually adjusting the intensity of the light to make it brighter. As the light intensity increases, I want you to tell me to stop when you cannot take it any more. It is important that at all times during this test you look straight ahead, and do not squint or close your eyes throughout this test. Do you have any questions?”

Technician: “Let me repeat the important points once again:

1. Look straight ahead at the blinking point at all times, and always keep your eyes open, whether the light is on or off.

2. When you can no longer tolerate the light, please tell me to stop.”

*Technician conducts VPTol assessment.*

Technician: “Rate the maximum pain intensity experienced during the previous test using a sliding scale from none to the most imaginable. Please do the same with unpleasantness.”

*Participant rates UP and PI for VPTol test.*

Technician: “Excellent, that concludes this final test and testing for the day. Thank you so much for your time!”
