# Supplementary material for: Assessment of light sensitivity using the Ocular Photosensitivity Analyzer: Discomfort threshold, pain threshold, and tolerance in healthy volunteers
Source: PLoS One. 2026 Oct 29;21(10):e0361637. doi: 10.1371/journal.pone.0361637 (PMC13718949; doi:10.1371/journal.pone.0361637)
Supplement: Supplementary file 3 [file pone.0361637.s004.docx]

**Supplementary Table S2.** **Shapiro-Wilk test for normality for all outcome variables**. One participant completed full OPA assessment but did not enter VAS ratings for UP and PI in the Tol test. A separate participant had no VPPT measured illuminance value due to technical error recording it, but otherwise completed full OPA assessment. This participant’s VAS ratings for the VPPT test were present and included in the analysis.

| **Variable** | **Shapiro-Wilk Statistic** | **df** | **p-value** |
| --- | --- | --- | --- |
| **Measured illuminance** |  |  |  |
| VPDT | 0.959 | 29 | 0.379 |
| VPPT | 0.952 | 28 | 0.223 |
| VPTol | 0.932 | 29 | 0.079 |
| **Unpleasantness** |  |  |  |
| VPDT | 0.897 | 29 | 0.005 |
| VPPT | 0.956 | 29 | 0.197 |
| VPTol | 0.972 | 28 | 0.641 |
| **Pain intensity** |  |  |  |
| VPDT | 0.689 | 29 | <0.001 |
| VPPT | 0.913 | 29 | 0.018 |
| VPTol | 0.783 | 28 | <0.001 |

df = Degrees of Freedom
