# Supplementary material for: Assessment of light sensitivity using the Ocular Photosensitivity Analyzer: Discomfort threshold, pain threshold, and tolerance in healthy volunteers
Source: PLoS One. 2026 Oct 29;21(10):e0361637. doi: 10.1371/journal.pone.0361637 (PMC13718949; doi:10.1371/journal.pone.0361637)
Supplement: Supplementary file 4 [file pone.0361637.s005.docx]

**Supplementary Table S3: Comorbidities and medications.** Displays comorbidities and medications for study participants. Medications taken by participants were stable and taken for ≥4 weeks. While not linked with alteration of pain perception, starting or stopping some of the included medications (e.g. systemic birth control) can affect mood or inflammatory background, and so in turn affect psychometric data.

|  | **Medication (Yes/No)** | **N** |
| --- | --- | --- |
| Birth control | Yes | 6 |
| Environmental allergy | Yes | 6 |
| Anxiety/Depression | Yes | 4 |
| Migraines | Yes | 2 |
| Acne | Yes | 1 |
| GAHT | Yes | 1 |
| Acid reflux | Yes | 1 |
| Medicated toothpaste | Yes | 1 |
| Vitamin D | Yes | 1 |
| Fish oil | Yes | 1 |
| Sleep apnea | No | 1 |
| Thyroid problems | No | 1 |

GAHT = gender affirming hormone therapy
