# Supplementary material for: Assessment of light sensitivity using the Ocular Photosensitivity Analyzer: Discomfort threshold, pain threshold, and tolerance in healthy volunteers
Source: PLoS One. 2026 Oct 29;21(10):e0361637. doi: 10.1371/journal.pone.0361637 (PMC13718949; doi:10.1371/journal.pone.0361637)
Supplement: Supplementary file 5 [file pone.0361637.s006.docx]

**Supplementary Table S4**. **Sex differences in measured illuminance values**. Mann-Whitney U tests to assess for sex differences in measured illuminance values, UP ratings, and PI ratings for VPDT, VPPT, and VPTol

| **Variable** | **Mann-Whitney U** | **Z** | **p-value** |
| --- | --- | --- | --- |
| **Unpleasantness** |  |  |  |
| VPDT | 98.500 | -0.284 | 0.780 |
| VPPT | 97.500 | -0.327 | 0.747 |
| VPTol | 92.000 | -0.253 | 0.821 |
| **Pain intensity** |  |  |  |
| VPDT | 78.000 | -1.204 | 0.252 |
| VPPT | 96.500 | -0.371 | 0.715 |
| VPTol | 96.500 | -0.047 | 0.964 |
