# Supplementary material for: Assessment of light sensitivity using the Ocular Photosensitivity Analyzer: Discomfort threshold, pain threshold, and tolerance in healthy volunteers
Source: PLoS One. 2026 Oct 29;21(10):e0361637. doi: 10.1371/journal.pone.0361637 (PMC13718949; doi:10.1371/journal.pone.0361637)
Supplement: Supplementary file 6 [file pone.0361637.s009.docx]

We thank the reviewers for this favorable and constructive review. Below, please find our point-by-point responses to the reviewer’s comments.

**Reviewer #1:**

**Title**: Appropriate

- n/a

**Abstract**: I would suggest that the authors make the abstract in a structured form.

- Per PLOS One formatting guidelines, we have left the abstract in the unstructured format.

**Introduction**: Authors have provided adequate literature and the justification for the study.

- n/a

**Methods**: Authors should provide the full name of YMCA's on first mention. Authors should clarify the reason behind the age range 18 to 39 years used as the criteria. Although, authors have indicated in the limitation section on the use of young adults, it would be helpful to indicate the reason.

- We have deleted the language regarding YMCA’s as it is fits well within the “community-based centers” later in the sentence on **line 106**.
- We have included “Participants were limited to young adults to minimize the effect of age-related ocular changes on light sensitivity measures” on **line 113** to provide rationale for our including only ages 18 to 39 years.

**Results**: No comment

- n/a

**Discussion**: The authors discussed the results and related literature well.

- n/a

**Reference**: there are some references that are very old as far as 1934. Are there no current studies on the nature of photophobia?

- The reference from 1934 was included as it was the first to describe photophobia. We include several other more recent references to provide perspective on how our understanding of photophobia has evolved from this initial definition in **paragraph 1** of the **introduction** section.

**Reviewer #2:** 

MAJOR COMMENTS
1. **The manuscript occasionally overstates what the study can demonstrate
The central finding, that participants reported distinguishable discomfort, pain, and intolerance levels during controlled light stimulation, is broadly supported by the presented data. However, the manuscript frequently extends beyond this observation into broader claims regarding:
i. multidimensional models of photophobia,
ii. diagnostic precision,
iii. pathophysiological mechanisms,
iv. clinical utility,
v. terminology reform.
These interpretations exceed what can reasonably be concluded from a relatively small cross-sectional psychophysical study involving 29 healthy volunteers.
Examples include:
• “These results support a multidimensional framework of photophobia”
• “may enhance diagnosis and treatment”
• “substantially improve clinical diagnosis”
• “advance in our understanding”
Such statements should be rewritten more cautiously and framed as preliminary, exploratory, or hypothesis-generating.
The present study demonstrates perceptual differentiation under a specific experimental paradigm. It does not establish diagnostic validity, mechanistic specificity, or clinical superiority.**

- We thank the reviewer for this point. We have revised the manuscript throughout to frame these broader implications as preliminary and have listed changes below, which include added text and edits to the 4 phrases referenced by the reviewer.

**Abstract**

- **Line 46:** *Although preliminary*, ~~T~~these ~~results~~ *findings are consistent with*~~support~~ a multidimensional framework of photophobia
- **Line 47:** Additionally, the OPA’s ability to differentially quantify VPDT, VPPT, and VPTol offers a novel approach to ~~objectively~~ characterize light sensitivity that may *aid future assessment* ~~enhance diagnosis and treatment~~ of abnormal photosensitivity.

**Introduction**

- **Line 92:** Quantifying these perceptual levels *is a first* ~~along the photophobia continuum marks an initial~~ step towards establishing healthy reference ranges, enables future OPA studies in patients with various conditions, and ~~is essential for integrating~~ *advances integration of* QST into investigations of light-induced pain, *which may in turn support further efforts to refine assessment and inform future therapeutic research* ~~thereby advancing diagnostic precision and informing the development of targeted therapies~~.

**Results and discussion**

- **Line 298:** The progression in measured illuminance across the tested perceptual levels provides ~~strong~~ *preliminary* evidence that light-induced pain ~~is not a~~ *may not be a* unitary phenomenon as originally described^2^, but rather exists along this perceptual continuum
- **Line 321:** By capturing and differentiating additional perceptual levels, our work ~~represents an advance in our understanding~~ *extends prior characterization* of light-induced discomfort and pain.
- **Line 324:** Our findings that VPPT exceeded VPDT and VPTol exceeded VPPT suggest a similar hierarchy exists *may exist* ~~exists~~ in light-induced pain.
- **Line 360:** Doing so may allow for a more comprehensive understanding of photophobia, *which may* ultimately ~~informing~~ *inform the development of* more precise diagnostic tools and targeted interventions for patients experiencing light sensitivity.
- **Line 365:** Though photophobia is known to exist along a continuum^5,6^, our work provides *preliminary* evidence ~~of~~ *suggesting* the existence of key points - discomfort, pain, and tolerance - along the range of light sensitivity that can be differentially activated in healthy subjects.
- **Line 380:** To address the gap between available terminology and these sensory experiences we ~~advocate for the use of~~ *propose, for consideration and further evaluation,* more precise terminology: “photodysesthesia” to denote sensations of abnormal discomfort induced by light, and “photoallodynia” to describe abnormal pain sensations triggered by light exposure.
- **Line 386:** Labelling light-induced pain during a normal eye exam as photoallodynia in a study of corneal neuropathy allowed for its isolation and subsequent treatment, confirming the term’s utility^45^ ~~.This gives credence to our argument~~ *and suggesting* that greater specification may lead to improved diagnosis and clinical outcomes.
- **Line 393:** *Further work is needed to clarify these proposed terms and validate their utility.*

**Conclusion**

- **Line 418:** The OPA’s ability to quantify distinct perceptual thresholds represents a ~~significant~~ methodological advance~~ment~~ in the assessment of light-induced pain.
- **Line 419:** It ~~is the first~~ *has the potential to be a* clinically suitable tool for QST of light sensitivity^1^ and further work should investigate its utility in this clinical setting.
- **Line 421:** Whereas further investigation is needed to establish normative reference ranges for these perceptual levels in healthy participants, the OPA may ~~greatly~~ enhance clinical trials by allowing for the evaluation of various sensory responses, including photodysesthesia and photoallodynia across various ophthalmic and neurologic pathologies.
- **Line 426:** Adopting more precise terminology and leveraging tools like the OPA *may eventually* ~~can substantially~~ improve clinical diagnosis, guide personalized treatments, and facilitate research into underlying pathophysiological mechanisms.

2. **The psychophysical constructs themselves require stronger validation
A major conceptual issue is that the study assumes participants can reliably and consistently distinguish between:
i. discomfort,
ii. pain,
iii. intolerance,
iv. unpleasantness,
v. pain intensity
as separable perceptual constructs.
However, these categories are inherently subjective, linguistically fluid, psychologically mediated, and potentially culturally variable. The manuscript provides no psychometric or construct validation demonstrating that participants interpret these concepts consistently.
This concern is particularly important because participants were explicitly instructed regarding distinctions between pain and unpleasantness, and these explanations may themselves shape participant responses.
Accordingly:
i. the observed differentiation may partly reflect instructional framing;
ii. semantic interpretation may vary substantially across participants;
iii. and the findings may not necessarily represent stable underlying perceptual constructs.
The manuscript should acknowledge these construct-validity limitations more explicitly and avoid implying that these perceptual categories are objectively discrete entities.**

We agree with the reviewer that we did not perform formal psychometric or construct validation and acknowledge the subjectivity in these terms. We have removed all mentions of the word “objective” in the manuscript. We also amended the limitations section by explicitly addressing the potential for subjects to have interpreted these concepts inconsistently.

We would also respectfully note one feature of the design relevant to this concern. The central finding of the graded separation in measured illuminance (VPDT < VPPT < VPTol) derives from a within-subjects paradigm in which each participant served as their own reference. A consistent rank ordering at the group level requires only that each individual apply the three criteria in an internally consistent order, not that participants share a common semantic definition of each term. Instructional framing and between-participant semantic variability therefore bear on the absolute values and cross-participant comparability of these constructs more than on the within-subject ordering that constitutes our primary result. We have added this clarification to the results and discussion section while still framing the broader construct interpretation as preliminary.

- **Line 288**: *While we did not formally assess whether participants interpreted the constructs of pain, discomfort, and tolerance uniformly*, ~~O~~our work *provides initial evidence* supporting~~s~~ these conceptual distinctions by showing that subjects can distinguish ~~discomfort from pain~~ them with instructions.
- **Line 305:** *Though the perceptual levels were operationally defined by task instructions, the within-subjects paradigm only required that each participants apply the instructions consistently, without assuming shared semantic interpretations across subjects. Thus*, ~~B~~*b*y providing discrete VPDT, VPPT, and VPTol values, the OPA offers an index of various points at which *an individual* ~~a person~~ may feel affected by light.
- **Line 399:** *We did not perform formal validation of the discomfort, pain, tolerance, unpleasantness, and pain intensity constructs, nor did we assess test-retest reliability. These categories are inherently subjective and the test instructions may have been interpreted differently by participants. Furthermore, the instructions* ~~E~~explicitly *defining pain versus unpleasantness are subject to the same variability in semantic understanding and* may have ~~influenced measured illuminance values and VAS ratings~~ *also contributed to the observed differentiations of perceptual levels and UP and PI.*
- **Line 411:** Future research should… “*establish test-retest reliability and construct validity for all terms subject to variation in semantic understanding,” …*

3. **VPTol methodology is fundamentally different from VPDT and VPPT
A major methodological issue is that VPTol was assessed using a substantially different protocol from VPDT and VPPT.
Specifically:
a. VPDT and VPPT used automated staircase procedures with reversal averaging;
b. VPTol used manually increased illumination over approximately 30 seconds;
c. VPTol was operator-controlled rather than algorithm-controlled;
d. VPTol was assessed only once per participant.
Consequently, VPTol may not represent a directly comparable psychophysical construct to VPDT and VPPT.
This introduces several important confounds, including:
i. temporal summation,
ii. adaptation effects,
iii. hysteresis effects,
iv. operator variability,
v. sequential conditioning,
vi. expectancy effects,
vii. and inconsistent stimulus kinetics.
Because of these methodological differences, direct quantitative comparison between VPTol and the other perceptual thresholds should be interpreted considerably more cautiously.
The manuscript acknowledges some of these issues in the Discussion; however, their implications are more substantial than currently presented.
At best, the authors should:
• explicitly discuss the non-equivalence of these paradigms;
• avoid implying direct comparability across all three measures;
• and clarify that VPTol remains exploratory.**

- We agree that the difference in methodology between assessments of VPDT and VPPT and VPTol limits the direct comparability across all three measures. Thus, we have added language throughout the discussion section clearly stating that the findings comparing the three levels must be interpreted with caution given the differences in assessments. Furthermore, we have clarified that VPTol remains exploratory. Finally, we have expanded on the limitations of manual VPTol assessment in the limitations section. In combination with the various other edits that substantially temper the conclusions drawn from our work, we feel that the below edits help to represent the limitations in implications drawn from assessment of and comparisons with VPTol.
- **Line 286**: *Though comparisons with VPTol are limited by the methodological differences from VPDT and VPDT testing*, ~~P~~participants exhibited a graduating trend in the respective measured illuminance values such that discomfort was less than pain and both were less than tolerance
- **Line 309**: *While it remains exploratory due to the operator-controlled ramp-up testing differing from the algorithm-controlled assessments of VPDT and VPPT*, ~~and thus~~ its inclusion extends the *potential* range of measurable responses and offers new insight into the wide range of light-induced pain.
- **Line 408**: Finally, manual adjustment during VPTol testing introduced ~~variability~~ *further* *confounders including temporal summation, adaptation, hysteresis, operator variability, expectancy, and inconsistent stimulus kinetics. Thus our VPTol findings and comparisons remain exploratory and must be interpreted with caution.*

4. **Sequential testing may have conditioned participant responses
All participants underwent testing in the same sequence:
a. VPDT
b. VPPT
c. VPTol
This fixed ordering introduces potential:
i. anticipation effects,
ii. habituation,
iii. sensitisation,
iv. perceptual anchoring,
v. learning effects,
vi. and carryover conditioning.
Participants were repeatedly exposed to escalating light stimuli while simultaneously being instructed regarding the expected perceptual categories. Consequently, later responses may have been influenced by prior exposure and expectation rather than reflecting fully independent perceptual phenomena.
This issue is particularly relevant in psychophysical studies where repeated exposure may alter threshold perception over time.
Although the rationale for the testing sequence is understandable, the lack of counterbalancing should be discussed more explicitly as an important limitation.**

- We agree that this should be explicitly noted as a limitation and have added the below language to the limitations section on **Line 406**: *The fixed testing order was not counterbalanced, confounding sequence with perceptual level. This may have also introduced anticipation, habituation, sensitization, anchoring, learning, or carryover effects during assessments.*

5. **Lack of test-retest reliability assessment
The manuscript repeatedly discusses the OPA as a quantitative sensory testing instrument suitable for clinical and research application. However, the present study provides no formal test-retest reliability assessment.
For psychophysical tools, reliability is fundamental.
The study would be substantially strengthened by reporting:
a. intra-session reliability,
b. repeatability coefficients,
c. intraclass correlation coefficients,
d. or threshold variability metrics.
Without such information, it remains difficult to determine whether the observed differences reflect stable perceptual phenomena or measurement variability.**

- We agree that test-retest reliability is important and have added language to the limitations section acknowledging this (See Reviewer 2, Major comment 2 for edits)

6. **Small sample size limits interpretability
The study includes only 29 participants.
While this may be acceptable for an exploratory psychophysical feasibility study, several interpretations are presented with a level of certainty more appropriate for a larger validation cohort.
Specific concerns include: limited statistical power, unstable subgroup analyses, inability to meaningfully assess sex differences and uncertain generalisability.
The manuscript should frame itself more explicitly as a preliminary or pilot investigation.**

- We have added language throughout the manuscript to frame our findings are preliminary (see Reviewer 2, Major comments 1, 2, and 3 for edits).

7. **The healthy volunteer population limits external validity
The study exclusively examined healthy young adults with minimal ocular pain and minimal photophobia symptoms.
Accordingly:
A. the findings cannot be extrapolated directly to clinical photophobia populations;
B. pathological photosensitivity mechanisms remain untested;
C. the proposed clinical implications remain speculative.
In addition, the highly controlled experimental environment which includes dark adaptation, repeated exposures, scripted instructions, and controlled LED stimulation, may not adequately reflect real-world photophobia experiences.
The manuscript should therefore avoid overstating translational or clinical applicability. This is important.**

- We agree with the reviewer that our prior language overstated the clinical applicability of our findings and have amended the manuscript accordingly (see Reviewer 2, Major comment 1 for edits).

8. **Statistical reporting requires major improvement. Several aspects of the statistical analysis require clarification or improvement.
a) One-tailed testing
The use of one-tailed paired t-tests requires stronger justification. Unless highly specific directional hypotheses were prespecified a priori, two-tailed testing would generally be more appropriate.
b) Assumptions of RM-ANOVA
The manuscript does not indicate whether:
i. sphericity assumptions were assessed;
ii. whether corrections (such as Greenhouse-Geisser adjustment)s were applied where necessary.
c) Effect sizes
The manuscript reports p-values extensively but provides limited effect size reporting outside ηp² for one analysis.
Additional effect size metrics would improve interpretability.
d) Multiple testing burden
A large number of post hoc comparisons were conducted in a relatively small cohort. While Holm correction was applied, the manuscript should acknowledge the increased risk of unstable estimates.**

- We thank the reviewer for bringing these concerns to our attention and have made changes accordingly.
- The language regarding one-tailed t-tests was an error left in from previous drafts, and the language has been corrected to reflect two-tailed t-tests, which is what the reported values are associated with. We removed the mention of one-tailed t-tests from the manuscript (**Line 248**).
- We re-ran the anovas and clarified assumptions of sphericity in the methods section (**Line 206**): *Sphericity was assessed for all within-subjects effects, and Greenhouse-Geisser-corrected degrees of freedom (ε = 0.85) are reported as a conservative measure.*
- We have added effect sizes for all the paired t tests, Friedman’s tests, and Wilcoxon’s tests.
- We added a section to the limitations section acknowledging the large number of post-hoc comparisons (**Line 404**): *Though Holm corrections were applied, the relatively small cohort (N=29) combined with the number of post-hoc comparisons increases the risk of unstable estimates.*

9. **The distinction between “pain” and “unpleasantness” may not be psychometrically robust
Participants were explicitly instructed regarding distinctions between pain intensity and unpleasantness. However:
a. the distinction may not have been uniformly understood;
b. repeated explanations may have introduced demand characteristics;
c. and the instructional framing itself may have shaped participant responses.
Accordingly, conclusions regarding “affective skewing” should be interpreted cautiously.
The current wording occasionally implies stronger mechanistic interpretation than the data justify.**

- We have shifted the section subheading to strike “~~affective skewing~~” in favor of “*greater UP than PI*” (**Line** **329**). We have also tempered the language stating our conclusions from these findings (see Reviewer 2, Major Comment 1 for edits)

10. **The terminology proposal section is excessively speculative. Section 4.3 which advocates “photodysesthesia” and “photoallodynia” extends well beyond the actual empirical findings of the study.
While the conceptual discussion is intellectually interesting, the present dataset does not directly validate these proposed diagnostic or phenomenological constructs.
This section currently reads more as a conceptual position paper than an evidence-based interpretation of the experimental results. The authors should substantially shorten and temper this section. The proposed terminology framework is not empirically validated by the present study and appears disproportionate to the scope of the findings.**

- We have added language clarifying the preliminary nature of our work (**Line 365**) and deleted a large portion of this section detailing the history of defining photophobia (**Line 369-376**), which we acknowledge provides more detail that necessary to convey the proposed terms. We further explicitly state that we are simply proposing these terms (**Line 381**) and that further work is needed to validate them and confirm their utility (**Line 393**).

11. **Potential conflict-of-interest implications should be handled with greater balance
Four of the authors are co-inventors of the OPA technology and may receive royalties from commercialisation.
The disclosure itself is appropriate and appreciated. However, portions of the Discussion and “Implications and future directions” section occasionally read more as advocacy for the device than objective evaluation.
The manuscript should ensure that:
i. claims regarding clinical applicability remain conservative;
ii. limitations are discussed with equal emphasis;
ii. the tone remains appropriately balanced.**

- We thank the reviewer for raising this point and have amended the conclusion section accordingly to temper our language (see Reviewer 2, Major comment 1 for edits)

**MINOR COMMENTS**
1. **Clarify participant ocular status
Although major ocular disease was excluded, the manuscript does not clearly state whether participants underwent:
a. visual acuity testing,
b. slit-lamp examination,
c. refractive assessment,
d. tear-film evaluation,
e. formal ocular surface examination.
This should be clarified.**

- We edited **Line 127** to reflect: These criteria were evaluated based on self-report during pre-screening*; participants did not undergo examinations or testing for study purposes*. ~~p~~Participants that met the criteria were considered healthy for the purposes of this study.

2. **Clarify testing conditions more explicitly
The Methods section should specify:
a. whether testing was monocular or binocular;
b. ambient lighting conditions;
c. fixation requirements;
d, whether pupil size was monitored or controlled.**

- We have clarified a), b), and d) in **Line 135**: *Testing was binocular and conducted in a dark room to minimize effects of light not produced by the OPA. Pupil size was not monitored.*
- c) is detailed in QST script in S1 protocol, which includes instructions throughout the assessments, such as “look straight ahead and focus on the white blinking point”

3. **Improve terminology consistency
The manuscript alternates between:
i. “pain threshold”,
ii. “light-induced pain”,
iii. “photosensitivity”,
iv. “discomfort threshold”,
v. “photophobia”.
Terminology should be standardised where possible.**

- We have standardized our terminology by only using “visual photosensitivity” in relation to the OPA, while using “light sensitivity” as the broader term in non-OPA contexts throughout the manuscript.
- We respectfully note that “discomfort threshold,” “pain threshold”, “light-induced pain,” and “photophobia” are not interchangeable. The idea that they denote distinct, but related constructs central to the manuscript: the first two are specific measured thresholds, "light-induced pain" is the broader phenomenon, and "photophobia" is the umbrella clinical term we argue is imprecise. We feel that standardizing these terms would limit these distinctions.

4. **Figure clarity
Figures 3 and 4 are somewhat difficult to interpret visually in their current form. Improved labelling, clearer legends, and more transparent depiction of distributional characteristics would improve readability.**

- We have attempted to clarify all of these characteristics for both figures along with the additional changes mentioned in Reviewer 2, minor comment 5.

5. **Reporting of VAS data
The manuscript alternates between means ± SD and medians with IQRs. While statistically justified, figure presentation should more clearly reflect the underlying distribution type.**

- Figure 3 was switched to a bar chart with means and error bars representing the SD to better reflect the way the data is reported in the manuscript.
- Figure 4 was switched to a box-and-whisker plot to better reflect the way most of the rating data is reported as median (IQR) in the manuscript. To further clarify the data visualization, we included the following in **Line 263**: *All UP and PI ratings are reported as median and IQR in* ***Fig 4****.*

6. **Language and style
Several sentences in the Discussion are unnecessarily long and occasionally repetitive. Some claims could be rewritten more concisely and neutrally.
Examples include:
“marks an initial step towards…”
“substantially improve clinical diagnosis…”
“advance in our understanding…”
These could be simplified to improve precision and reduce interpretive overreach.**

- We have attempted to decrease repetitive language throughout the manuscript. Several of these examples were already edited when addressing other comments and the remaining “marks an initial step towards” has been edited appropriately (**Line 92**): Quantifying these perceptual levels *is a first* ~~along the photophobia continuum marks an initial~~ step towards establishing healthy reference ranges…
