# Supplementary material for: Assessment of light sensitivity using the Ocular Photosensitivity Analyzer: Discomfort threshold, pain threshold, and tolerance in healthy volunteers
Source: PLoS One. 2026 Oct 29;21(10):e0361637. doi: 10.1371/journal.pone.0361637 (PMC13718949; doi:10.1371/journal.pone.0361637)
Supplement: Supplementary file 7 [file pone.0361637.s010.docx]

**Comments to the authors**

This manuscript evaluates discomfort threshold, pain threshold, and tolerance to light using the Ocular Photosensitivity Analyzer in 29 healthy young adults. The topic is relevant, and the study may provide useful exploratory evidence that participants identify a higher illuminance as painful than as uncomfortable. However, several methodological and reporting issues currently prevent the central conclusions from being fully supported.

**Major comments**

**1. The three outcomes were not measured using equivalent procedures.**
VPDT and VPPT were assessed with an automated staircase and calculated from ten response reversals. VPTol was measured once using an operator-controlled gradual ramp. Therefore, the VPPT–VPTol difference is confounded by stimulus delivery, duration, operator involvement, and measurement reliability. VPTol should be treated as a separate exploratory outcome rather than as an equivalent third level in a validated three-level continuum. The abstract, Results, Discussion, and Conclusions should be revised accordingly.

**2. Perceptual level is confounded with test order.**
All participants completed VPDT, VPPT, and VPTol in that fixed sequence. Consequently, the observed ordering may partly reflect anchoring, expectancy, learning, sensitization, or habituation. The study cannot distinguish these order effects from differences among the constructs. This should be made explicit, and statements that the study demonstrates three distinct sensory experiences should be substantially moderated.

**3. A Discussion statement contradicts the statistical results.**
The Results state that VPTol pain intensity was not significantly greater than VPDT pain intensity (p=0.065) and that VPTol did not differ significantly from VPPT in either pain intensity or unpleasantness. The Discussion nevertheless states that both unpleasantness and pain intensity were significantly greater for VPTol and VPPT than for VPDT. Please correct this statement and verify all corresponding claims in the abstract, figure legends, and conclusion.

**4. Statistical reporting and missing-data handling require clarification.**
Please report the exact sample size included in every omnibus and pairwise analysis, explain the varying degrees of freedom, and state how missing observations were handled. The reported Mauchly result is incomplete—“Mauchly’s W=0.82, p )”—and should be corrected. Please also ensure that Holm-adjusted p-values are identified consistently and that confidence intervals are provided for the primary differences where feasible.

**5. The interpretation should remain exploratory.**
The study did not establish construct validity, test–retest reliability, clinical diagnostic performance, or abnormal reference ranges. The data show that participants selected different light levels in response to different instructions, with VPTol additionally assessed using a different procedure. They do not yet validate discomfort, pain, and tolerance as three distinct and valid OPA constructs. The proposed terms “photodysesthesia” and “photoallodynia” may remain as hypotheses for future research, but they should not be presented as empirically validated by this dataset.

**Minor comments**

1. Report non-normally distributed VAS outcomes consistently as medians and IQRs. The current text mixes median/IQR reporting with values formatted as mean ± SD.
2. Describe participants as individuals without relevant self-reported conditions, because no formal ocular examinations were performed.
3. Provide sufficient technical information for replication, including the photometer manufacturer and model, calibration approach, stimulus geometry, and the procedure and approximate rate used for the manual VPTol ramp.
4. Clarify whether the clear lenses were plano lenses and whether lens material, coating, and other relevant properties were standardized across participants.
5. State whether any participant reached the device’s maximum output before reporting pain or intolerance and explain how such observations were handled analytically.
6. Explain how the instruction to indicate intolerance “without squinting” was operationalized, how squinting was monitored, and what occurred if a participant squinted involuntarily.
7. Provide de-identified participant-level data and, preferably, analysis syntax. Figures should show the paired individual observations rather than only aggregate bars or box plots.
8. Correct the following textual issues: “VPTol exceed VPPT” should be “VPTol exceeded VPPT”; “differences from VPDT and VPDT testing” presumably should read “differences from VPDT and VPPT testing”; and “Helsinki Accord” should be replaced with “Declaration of Helsinki.”
9. Explain why assent is mentioned when all analyzed participants were aged 19–34 years.
10. Consider separating Results and Discussion. At minimum, distinguish direct findings clearly from interpretation.

**Recommendation: MAJOR REVISION**

The study has publishable exploratory potential, but its conclusions must be brought into closer alignment with the procedures and statistical findings. In particular, VPTol should be presented as an exploratory outcome, the implications of the fixed testing order should be clearly acknowledged, and the statistical and reporting inconsistencies should be corrected. The manuscript may become suitable for publication if the authors adequately address these issues and substantially moderate claims that cannot be supported by the present design.
