# Supplementary material for: Assessment of light sensitivity using the Ocular Photosensitivity Analyzer: Discomfort threshold, pain threshold, and tolerance in healthy volunteers
Source: PLoS One. 2026 Oct 29;21(10):e0361637. doi: 10.1371/journal.pone.0361637 (PMC13718949; doi:10.1371/journal.pone.0361637)
Supplement: Supplementary file 8 [file pone.0361637.s011.docx]

**Response to Reviews**

We thank the reviewers for their time and thoughtful evaluation of our manuscript. As noted in their comments, no further revisions were requested. We provide formal responses below to comply with the journal’s resubmission requirements.

**Reviewer 1**

**Comment:** “All concerns have been addressed; the manuscript is recommended for publication.”

**Response:** We appreciate the reviewer’s careful assessment and positive recommendation. No additional revisions were requested, and no changes have been made to the manuscript.

**Reviewer 2**

**Comment:** “The authors have satisfactorily addressed all comments.”

**Response:** We thank the reviewer for their supportive evaluation. As no further modifications were requested, the manuscript remains unchanged.

**Summary of Revisions**

No revisions were required or made. A tracked‑changes version has been provided containing a brief note indicating that no changes were requested, to satisfy system requirements.
