# Supplementary material for: Assessment of light sensitivity using the Ocular Photosensitivity Analyzer: Discomfort threshold, pain threshold, and tolerance in healthy volunteers
Source: PLoS One. 2026 Oct 29;21(10):e0361637. doi: 10.1371/journal.pone.0361637 (PMC13718949; doi:10.1371/journal.pone.0361637)
Supplement: Supplementary file 9 [file pone.0361637.s012.docx]

We thank the reviewer for their insightful comments. Below, please find our point-by-point. Added text is underlined and removed text is indicated by ~~strikethrough~~. Line numbers correspond to the text’s location in the file named “Manuscript”.

**Reviewer #2: Major comments**

| **1. The three outcomes were not measured using equivalent procedures.**  VPDT and VPPT were assessed with an automated staircase and calculated from ten response reversals. VPTol was measured once using an operator-controlled gradual ramp. Therefore, the VPPT–VPTol difference is confounded by stimulus delivery, duration, operator involvement, and measurement reliability. VPTol should be treated as a separate exploratory outcome rather than as an equivalent third level in a validated three-level continuum. The abstract, Results, Discussion, and Conclusions should be revised accordingly. | We acknowledge that the three outcomes were not measured using equivalent procedures. This was a result of limitations in the OPA’s software at the time of data collection, as automated VPTol assessment was not possible. As such, we understand that outcomes of VPTol assessments should be discussed in a more exploratory manner and have made the following edits to various sections of the manuscript to better reflect this change.  **Abstract**  **Line** **31**: … to investigate three perceptual levels in healthy adults: visual photosensitivity discomfort threshold (VPDT), visual photosensitivity pain threshold (VPPT), and an exploratory outcome, visual photosensitivity tolerance level (VPTol).  **Results**  **Line 237:** indicating QST using the OPA successfully differentiated between VPDT, VPPT, and in an exploratory manner, VPTol in healthy participants.  **Line 239**: … and though not a direct comparison, VPTol values were significantly greater than both those for VPPT  **Discussion**  **Line 283**: Though comparisons with VPTol are limited by the methodological differences from VPDT and VPPDT testing,  **Line 286**: While we did not formally assess whether participants interpreted the constructs of pain, discomfort, and tolerance uniformly, our work provides initial evidence supporting these conceptual distinctions by showing that subjects can distinguish them with instructions  **Line 306:** While it remains exploratory due to the operator-controlled ramp-up testing differing from the algorithm-controlled assessments of VPDT and VPPT, its inclusion extends the potential range of measurable responses and offers new insight into the wide range of light-induced pain.  **Line 361:** Though photophobia is known to exist along a continuum^5,6^, our work provides preliminary evidence suggesting the existence of key points - discomfort, pain, and tolerance - along the range of light sensitivity that can be differentially activated in healthy subjects.  **Line 395**: Finally, manual adjustment during VPTol testing introduced further confounders including temporal summation, adaptation, hysteresis, operator variability, expectancy, and inconsistent stimulus kinetics. Additionally, squinting was not monitored and the VPTol test did not stop if a participant squinted involuntary. Thus our VPTol findings and comparisons remain exploratory and must be interpreted with caution. |
| --- | --- |
| **2. Perceptual level is confounded with test order.**  All participants completed VPDT, VPPT, and VPTol in that fixed sequence. Consequently, the observed ordering may partly reflect anchoring, expectancy, learning, sensitization, or habituation. The study cannot distinguish these order effects from differences among the constructs. This should be made explicit, and statements that the study demonstrates three distinct sensory experiences should be substantially moderated. | We agree that the fixed sequences of assessments is a limitation of our work and may have led to confounding effects. To acknowledge this, we have explicitly stated the following text in the in the **Limitations** section (**Line 392)**:  “The fixed testing order was not counterbalanced, confounding sequence with perceptual level. This may have also introduced anticipation, habituation, sensitization, anchoring, learning, or carryover effects during assessments”  See revisions in **Major comment #1** for further revisions tempering conclusions and emphasizing the preliminary, exploratory nature of the results. |
| **3. A Discussion statement contradicts the statistical results.**  The Results state that VPTol pain intensity was not significantly greater than VPDT pain intensity (p=0.065) and that VPTol did not differ significantly from VPPT in either pain intensity or unpleasantness. The Discussion nevertheless states that both unpleasantness and pain intensity were significantly greater for VPTol and VPPT than for VPDT. Please correct this statement and verify all corresponding claims in the abstract, figure legends, and conclusion. | We thank the reviewer for catching this error and apologize for the mistake. The sentence was incorrect and has now been corrected by removal.  All other corresponding claims throughout the manuscript reported these results correctly. |
| **4. Statistical reporting and missing-data handling require clarification.**  Please report the exact sample size included in every omnibus and pairwise analysis, explain the varying degrees of freedom, and state how missing observations were handled. The reported Mauchly result is incomplete—“Mauchly’s W=0.82, p )”—and should be corrected. Please also ensure that Holm-adjusted p-values are identified consistently and that confidence intervals are provided for the primary differences where feasible. | We appreciate the reviewer’s observation and agree that the statistical reporting in the manuscript would benefit from further clarification.   Sample sizes have been included in the parentheses for all omnibus and pairwise analyses as “N=”.  The following language has been included in **Line 224** to explain the varying degrees of freedom among the different analyses, which were due to missing observations:  “One participant reached the OPA’s maximum output during the VPPT test and their lux value was excluded analytically, though the UP and PI values were included. A separate participant completed VPTol assessment, but did not record UP or PI values, and thus only their lux value was included in analysis”  We apologize for this accidental exclusion and the p-value for Mauchly’s W has now been included in the abstract (**Line 37**) and results (**Line 235**)  Holm-adjusted p-values have now been specified as p_adj (they were previously labeled as “p”).  Furthermore, confidence intervals have been provided for post-hoc t-tests for perceptual level. With the SPSS version utilized, they were not calculable for the Wilcoxon tests for VAS ratings. |
| **5. The interpretation should remain exploratory.**  The study did not establish construct validity, test–retest reliability, clinical diagnostic performance, or abnormal reference ranges. The data show that participants selected different light levels in response to different instructions, with VPTol additionally assessed using a different procedure. They do not yet validate discomfort, pain, and tolerance as three distinct and valid OPA constructs. The proposed terms “photodysesthesia” and “photoallodynia” may remain as hypotheses for future research, but they should not be presented as empirically validated by this dataset. | We agree that the interpretation should remain exploratory; the study’s purpose was to examine whether the OPA could distinguish between these perceptual levels, but did not formally validate them as separate constructs, which is an important limitation. Thus, we have included language in the **Limitations** section to mention these weaknesses related to validation, lack of test-retest reliability, and the subjective nature of the instructions.  **Lines** **386:** “We did not perform formal validation of the discomfort, pain, tolerance, unpleasantness, and pain intensity constructs, nor did we assess test-retest reliability. These categories are inherently subjective and the test instructions may have been interpreted differently by participants.”  Furthermore, we agree that the proposed terms are not validated, and the goal of discussing them was to introduce them as ideas for future research to explore further. Thus, we have included language in the **Discussion** section to highlight this goal and clarify that the terms are merely hypotheses for future work at this time.  **Line 368:** “To address the gap between available terminology and these sensory experiences we propose, for consideration and further evaluation, more precise terminology: “photodysesthesia” to denote sensations of abnormal discomfort induced by light, and “photoallodynia” to describe abnormal pain sensations triggered by light exposure.”  See revisions in **Major comment #1** for further revisions tempering conclusions and emphasizing the preliminary, exploratory nature of the results. |

**Reviewer #2: Minor comments**

| 1. Report non-normally distributed VAS outcomes consistently as medians and IQRs. The current text mixes median/IQR reporting with values formatted as mean ± SD. | We agree that consistency in reporting these values is important. As such, we have reported all VAS outcomes as medians with IQRs (**Line 256**) and added a relevant line clarifying this in the **Methods** (**Line 209**). |
| --- | --- |
| 2. Describe participants as individuals without relevant self-reported conditions, because no formal ocular examinations were performed. | We acknowledge the importance of making this clarification and have made the following edits.  We also added the below language to the Limitations section in **Line 383**:  Limitations of this study include the use of only young ~~healthy~~ volunteers whose conditions were self-reported and no formal ocular examinations were performed. Thus they ~~who~~ may have exhibited different responses than different age groups and clinical populations experiencing abnormal light sensitivity.  Additionally, the below language is included in the **Methods** section in **Line 120**:  “These criteria were evaluated based on self-report during pre-screening; participants did not undergo examinations or testing for study purposes.” |
| 3. Provide sufficient technical information for replication, including the photometer manufacturer and model, calibration approach, stimulus geometry, and the procedure and approximate rate used for the manual VPTol ramp. | We thank the reviewer for this observation and have provided information for the photometer below.  The photometer information is presented in **line** **153.**  “The light intensity at that point was measured with a ~~photometer~~ digital lux meter (LX1010B, Dr. meter, Thousandshores Inc, Union City, CA) to determine VPTol.  We have presented language to detail the protocol for the manual VPTol ramp as listed below. (**Lines 148-150):**  “To evaluate VPTol, rather than the OPA increasing light intensity in discrete steps in an automated manner, instead light intensity was manually increased by the operator in a gradual manner such that it would require approximately 30 seconds to reach the OPA’s maximum output of 4.5 log lux.” |
| 4. Clarify whether the clear lenses were plano lenses and whether lens material, coating, and other relevant properties were standardized across participants. | We have made the following clarification in **Line 159:**  Clear lenses were plano lenses and standardized across participants. |
| 5. State whether any participant reached the device’s maximum output before reporting pain or intolerance and explain how such observations were handled analytically. | There was one participant who reached the OPA’s maximum output during a VPPT assessment. No other subject’s reached the maximum output during any other assessments. While the OPA could not calculate the exact lux value for the VPPT, it was able to output enough lux such that the participant reported pain. Thus, we excluded the VPPT lux value from the results as it was provided to us by the OPA as “35000+”, but included the VAS ratings for UP and PI. Language explaining our handling of this participant’s data is included in **Line 224:**  “One participant reached the OPA’s maximum output during the VPPT test and their lux value was excluded analytically, though the UP and PI values were included.” |
| 6. Explain how the instruction to indicate intolerance “without squinting” was operationalized, how squinting was monitored, and what occurred if a participant squinted involuntarily | We thank the reviewer for observing this missing clarification. We describe how squinting was operationalized in the following language from the VPTol Trial in **S1 Protocol**:  “It is important that at all times during this test you look straight ahead, and do not squint or close your eyes throughout this test….Look straight ahead at the blinking point at all times, and always keep your eyes open, whether the light is on or off….When you can no longer tolerate the light, please tell me to stop.”  However, we did not monitor squinting and did not conclude testing if a participant squinted. As such, we acknowledge that our handling of squinting may have res  ulted in a elevated threshold values for participants and have added the following clarifications to the **Methods** and **Limitations**:  **Line 152:** “Squinting was not monitored and the test did not stop if a participant squinted involuntary.”  **Line 397**: “Additionally, squinting was not monitored and the VPTol test did not stop if a participant squinted involuntary.” |
| 7. Provide de-identified participant-level data and, preferably, analysis syntax. Figures should show the paired individual observations rather than only aggregate bars or box plots. | We agree that this information would be helpful and have provided participant-level de-identified data in the excel file labeled “Data.”  We have included the analysis syntax in a word document file labeled “Analysis Syntax”, which corresponds to the excel labeled “Data_formatted_for_analysis”.  We appreciate this suggestion and have re-worked **Fig 3** and **Fig 4** to show the individual observations as well as the aggregate bars and plots. The phrase “Individual participant ratings are plotted as dots.” has also been added to both figure descriptions accordingly. |
| 8. Correct the following textual issues: “VPTol exceed VPPT” should be “VPTol exceeded VPPT”; “differences from VPDT and VPDT testing” presumably should read “differences from VPDT and VPPT testing”; and “Helsinki Accord” should be replaced with “Declaration of Helsinki.” | We have made the following corrections:  1. **Line 38:** exceed -> exceeded  2. **Line 284**: VPDT->VPPT  3. **Line 97:** Helsinki Accord -> Declaration of Helsinki |
| 9. Explain why assent is mentioned when all analyzed participants were aged 19–34 years | This was a mistake – all participants provided informed consent before any study procedures began and we have removed “or assent”. |
| 10. Consider separating Results and Discussion. At minimum, distinguish direct findings clearly from interpretation. | We had originally combined the two sections per the PLOS One instructions for authors. However, we agree there would be benefit in distinguishing the two and have added a separate heading for “Discussion” on **Line 279**. |
