# Supplementary material for: Assessment of light sensitivity using the Ocular Photosensitivity Analyzer: Discomfort threshold, pain threshold, and tolerance in healthy volunteers
Source: PLoS One. 2026 Oct 29;21(10):e0361637. doi: 10.1371/journal.pone.0361637 (PMC13718949; doi:10.1371/journal.pone.0361637)
Supplement: Peer review history

# Peer review: Decision Letter 0

**DOI:** 10.1371/journal.pone.0361637.r001

**Authors:** Kofi Asiedu

11 May 2026

Dear Dr. Moulton,

Thank you for submitting your manuscript to PLOS ONE. After careful consideration, we feel that it has merit but does not fully meet PLOS ONE’s publication criteria as it currently stands. Therefore, we invite you to submit a revised version of the manuscript that addresses the points raised during the review process.

Please submit your revised manuscript by Jun 25 2026 11:59PM. If you will need more time than this to complete your revisions, please reply to this message or contact the journal office at plosone@plos.org. When you're ready to submit your revision, log on to https://www.editorialmanager.com/pone/ and select the 'Submissions Needing Revision' folder to locate your manuscript file.

- A letter that responds to each point raised by the academic editor and reviewer(s). You should upload this letter as a separate file labeled 'Response to Reviewers'.
- A marked-up copy of your manuscript that highlights changes made to the original version. You should upload this as a separate file labeled 'Revised Manuscript with Track Changes'.
- An unmarked version of your revised paper without tracked changes. You should upload this as a separate file labeled 'Manuscript'.

If you would like to make changes to your financial disclosure, please include your updated statement in your cover letter. Guidelines for resubmitting your figure files are available below the reviewer comments at the end of this letter.

If applicable, we recommend that you deposit your laboratory protocols in protocols.io to enhance the reproducibility of your results. Protocols.io assigns your protocol its own identifier (DOI) so that it can be cited independently in the future. For instructions see: https://journals.plos.org/plosone/s/submission-guidelines#loc-laboratory-protocols. Additionally, PLOS ONE offers an option for publishing peer-reviewed Lab Protocol articles, which describe protocols hosted on protocols.io. Read more information on sharing protocols at https://plos.org/protocols?utm_medium=editorial-email&utm_source=authorletters&utm_campaign=protocols.

As the corresponding author, your ORCID iD is verified in the submission system and will appear in the published article. PLOS supports the use of ORCID, and we encourage all coauthors to register for an ORCID iD and use it as well. Please encourage your coauthors to verify their ORCID iD within the submission system before final acceptance, as unverified ORCID iDs will not appear in the published article. *Only*  the individual author can complete the verification step; PLOS staff *cannot*  verify ORCID iDs on behalf of authors.

We look forward to receiving your revised manuscript.

Kind regards,

Kofi Asiedu, O.D., Ph.D. FAAO

Academic Editor

PLOS One

Journal Requirements:

When submitting your revision, we need you to address these additional requirements.

1. Please ensure that your manuscript meets PLOS ONE's style requirements, including those for file naming. The PLOS ONE style templates can be found at https://journals.plos.org/plosone/s/file?id=wjVg/PLOSOne_formatting_sample_main_body.pdf and https://journals.plos.org/plosone/s/file?id=ba62/PLOSOne_formatting_sample_title_authors_affiliations.pdf

2. We note that the grant information you provided in the ‘Funding Information’ and ‘Financial Disclosure’ sections do not match.

When you resubmit, please ensure that you provide the correct grant numbers for the awards you received for your study in the ‘Funding Information’ section.

3. Thank you for stating the following financial disclosure:

[The author(s) received no specific funding for this work.].

At this time, please address the following queries:

a) Please clarify the sources of funding (financial or material support) for your study. List the grants or organizations that supported your study, including funding received from your institution.

b) State what role the funders took in the study. If the funders had no role in your study, please state: “The funders had no role in study design, data collection and analysis, decision to publish, or preparation of the manuscript.”

c) If any authors received a salary from any of your funders, please state which authors and which funders.

d) If you did not receive any funding for this study, please state: “The authors received no specific funding for this work.”

Please include your amended statements within your cover letter; we will change the online submission form on your behalf.

4. Thank you for stating the following in your manuscript:

[Funding for this project was supported by the Mayday/Herlands Chair Fund (EAM) and startup funds from the Department of Anesthesiology, Critical Care and Pain Medicine, Boston Children’s Hospital (EAM).]

We note that you have provided funding information that is not currently declared in your Funding Statement. However, funding information should not appear in the Acknowledgments section or other areas of your manuscript. We will only publish funding information present in the Funding Statement section of the online submission form.

Please remove any funding-related text from the manuscript and let us know how you would like to update your Funding Statement. Currently, your Funding Statement reads as follows:

[The author(s) received no specific funding for this work.]

Please include your amended statements within your cover letter; we will change the online submission form on your behalf.

5. We note that you have a patent relating to material pertinent to this article. Please provide an amended statement of Competing Interests to declare this patent (with details including name and number), along with any other relevant declarations relating to employment, consultancy, patents, products in development or modified products etc. Please confirm that this does not alter your adherence to all PLOS ONE policies on sharing data and materials, as detailed online in our guide for authors http://journals.plos.org/plosone/s/competing-interests by including the following statement: "This does not alter our adherence to PLOS ONE policies on sharing data and materials.” If there are restrictions on sharing of data and/or materials, please state these. Please note that we cannot proceed with consideration of your article until this information has been declared.

Please respond by return email and we will update the online submission form on your behalf.

6. We note that Figure 1 in your submission contains images which may be copyrighted. All PLOS content is published under the Creative Commons Attribution License (CC BY 4.0), which means that the manuscript, images, and Supporting Information files will be freely available online, and any third party is permitted to access, download, copy, distribute, and use these materials in any way, even commercially, with proper attribution. For more information, see our copyright guidelines: http://journals.plos.org/plosone/s/licenses-and-copyright.

We require you to either (1) present written permission from the copyright holder to publish these figures specifically under the CC BY 4.0 license, or (2) remove the figures from your submission:

1. You may seek permission from the original copyright holder of Figure 1 to publish the content specifically under the CC BY 4.0 license.

We recommend that you contact the original copyright holder with the Content Permission Form (http://journals.plos.org/plosone/s/file?id=7c09/content-permission-form.pdf) and the following text:

“I request permission for the open-access journal PLOS ONE to publish XXX under the Creative Commons Attribution License (CCAL) CC BY 4.0 (http://creativecommons.org/licenses/by/4.0/). Please be aware that this license allows unrestricted use and distribution, even commercially, by third parties. Please reply and provide explicit written permission to publish XXX under a CC BY license and complete the attached form.”

Please upload the completed Content Permission Form or other proof of granted permissions as an "Other" file with your submission.

In the figure caption of the copyrighted figure, please include the following text: “Reprinted from [ref] under a CC BY license, with permission from [name of publisher], original copyright [original copyright year].”

2. If you are unable to obtain permission from the original copyright holder to publish these figures under the CC BY 4.0 license or if the copyright holder’s requirements are incompatible with the CC BY 4.0 license, please either i) remove the figure or ii) supply a replacement figure that complies with the CC BY 4.0 license. Please check copyright information on all replacement figures and update the figure caption with source information. If applicable, please specify in the figure caption text when a figure is similar but not identical to the original image and is therefore for illustrative purposes only.

7. We note that there is identifying data in Table 1. Prior to sharing human research participant data, authors should consult with an ethics committee to ensure data are shared in accordance with participant consent and all applicable local laws.

Data sharing should never compromise participant privacy. It is therefore not appropriate to publicly share personally identifiable data on human research participants. The following are examples of data that should not be shared:

-Name, initials, physical address

-Ages more specific than whole numbers

-Internet protocol (IP) address

-Specific dates (birth dates, death dates, examination dates, etc.)

-Contact information such as phone number or email address

-Location data

-ID numbers that seem specific (long numbers, include initials, titled “Hospital ID”) rather than random (small numbers in numerical order)

Data that are not directly identifying may also be inappropriate to share, as in combination they can become identifying. For example, data collected from a small group of participants, vulnerable populations, or private groups should not be shared if they involve indirect identifiers (such as sex, ethnicity, location, etc.) that may risk the identification of study participants.

Additional guidance on preparing raw data for publication can be found in our Data Policy (https://journals.plos.org/plosone/s/data-availability#loc-human-research-participant-data-and-other-sensitive-data) and in the following article: http://www.bmj.com/content/340/bmj.c181.long.

Please remove or anonymize all personal information (sex, ethnicity, race), ensure that the data shared are in accordance with participant consent, and add an anonymized version to your manuscript file.

8. Please include captions for your Supporting Information files at the end of your manuscript, and update any in-text citations to match accordingly. Please see our Supporting Information guidelines for more information: http://journals.plos.org/plosone/s/supporting-information.

9. We note that there is identifying data in the Supporting Information file <OPA-Thresholds-Data.xlsx>. Due to the inclusion of these potentially identifying data, we have removed this file from your file inventory. Prior to sharing human research participant data, authors should consult with an ethics committee to ensure data are shared in accordance with participant consent and all applicable local laws.

Data sharing should never compromise participant privacy. It is therefore not appropriate to publicly share personally identifiable data on human research participants. The following are examples of data that should not be shared:

-Name, initials, physical address

-Ages more specific than whole numbers

-Internet protocol (IP) address

-Specific dates (birth dates, death dates, examination dates, etc.)

-Contact information such as phone number or email address

-Location data

-ID numbers that seem specific (long numbers, include initials, titled “Hospital ID”) rather than random (small numbers in numerical order)

Data that are not directly identifying may also be inappropriate to share, as in combination they can become identifying. For example, data collected from a small group of participants, vulnerable populations, or private groups should not be shared if they involve indirect identifiers (such as sex, ethnicity, location, etc.) that may risk the identification of study participants.

Additional guidance on preparing raw data for publication can be found in our Data Policy (https://journals.plos.org/plosone/s/data-availability#loc-human-research-participant-data-and-other-sensitive-data) and in the following article: http://www.bmj.com/content/340/bmj.c181.long.

Please remove or anonymize all personal information (specific dates, ID numbers that seem specific, location data, ethnicity, gender, race), ensure that the data shared are in accordance with participant consent, and re-upload a fully anonymized data set. Please note that spreadsheet columns with personal information must be removed and not hidden as all hidden columns will appear in the published file.

If the reviewer comments include a recommendation to cite specific previously published works, please review and evaluate these publications to determine whether they are relevant and should be cited. There is no requirement to cite these works unless the editor has indicated otherwise.

[Note: HTML markup is below. Please do not edit.]

Reviewers' comments:

Reviewer's Responses to Questions

**Comments to the Author**

1. Is the manuscript technically sound, and do the data support the conclusions?

Reviewer #1: Yes

Reviewer #2: Partly

**********

2. Has the statistical analysis been performed appropriately and rigorously?

Reviewer #1: Yes

Reviewer #2: No

**********

3. Have the authors made all data underlying the findings in their manuscript fully available?

The PLOS Data policy

Reviewer #1: Yes

Reviewer #2: Yes

**********

4. Is the manuscript presented in an intelligible fashion and written in standard English?

Reviewer #1: Yes

Reviewer #2: Yes

**********

Reviewer #1: Title: Appropriate

Abstract: I would suggest that the authors make the abstract in a structured form.

Introduction: Authors have provided adequate literature and the justification for the study.

Methods: Authors should provide the full name of YMCA's on first mention. Authors should clarify the reason behind the age range 18 to 39 years used as the criteria. Although, authors have indicated in the limitation section on the use of young adults, it would be helpful to indicate the reason.

Results: No comment

Discussion: The authors discussed the results and related literature well.

Reference: there are some references that are very old as far as 1934. Are there no current studies on the nature of photophobia?

Reviewer #2: Manuscript title: Assessment of light sensitivity using the Ocular Photosensitivity Analyzer: discomfort threshold, pain threshold, and tolerance in healthy volunteers

Recommendation: Major Revision

GENERAL COMMENTS

This manuscript evaluates the ability of the Ocular Photosensitivity Analyzer (OPA) to differentiate discomfort threshold, pain threshold, and tolerance to light in healthy volunteers using a quantitative sensory testing (QST) framework. The topic is clinically and scientifically relevant, particularly given the ongoing absence of standardised approaches for quantifying photophobia and light-induced pain. The manuscript is generally well organised, technically readable, and conceptually interesting. Applying QST principles to visual photosensitivity represents a potentially useful methodological direction.

Importantly, the study aligns with the broader aims of PLOS ONE by focusing primarily on technical feasibility and measurement characterisation rather than novelty alone. Ethical approval, data availability, and competing-interest disclosures are appropriately provided.

However, despite these strengths, several important methodological, psychophysical, analytical, and interpretive concerns currently limit confidence in the broader conclusions. In particular:

1. the sample size is small relative to the breadth of interpretation;

2. several conclusions extend beyond what the data can directly support;

3. the psychophysical constructs under investigation are insufficiently validated;

4. the methodology for VPTol differs fundamentally from that of VPDT and VPPT;

5. and portions of the Discussion drift from empirical interpretation into conceptual and terminological advocacy insufficiently supported by the presented findings.

The manuscript would therefore benefit substantially from methodological clarification, more cautious interpretation, and tighter alignment between the experimental data and the claims being advanced.

MAJOR COMMENTS

1. The manuscript occasionally overstates what the study can demonstrate

The central finding, that participants reported distinguishable discomfort, pain, and intolerance levels during controlled light stimulation, is broadly supported by the presented data. However, the manuscript frequently extends beyond this observation into broader claims regarding:

i. multidimensional models of photophobia,

ii. diagnostic precision,

iii. pathophysiological mechanisms,

iv. clinical utility,

v. terminology reform.

These interpretations exceed what can reasonably be concluded from a relatively small cross-sectional psychophysical study involving 29 healthy volunteers.

Examples include:

• “These results support a multidimensional framework of photophobia”

• “may enhance diagnosis and treatment”

• “substantially improve clinical diagnosis”

• “advance in our understanding”

Such statements should be rewritten more cautiously and framed as preliminary, exploratory, or hypothesis-generating.

The present study demonstrates perceptual differentiation under a specific experimental paradigm. It does not establish diagnostic validity, mechanistic specificity, or clinical superiority.

2. The psychophysical constructs themselves require stronger validation

A major conceptual issue is that the study assumes participants can reliably and consistently distinguish between:

i. discomfort,

ii. pain,

iii. intolerance,

iv. unpleasantness,

v. pain intensity

as separable perceptual constructs.

However, these categories are inherently subjective, linguistically fluid, psychologically mediated, and potentially culturally variable. The manuscript provides no psychometric or construct validation demonstrating that participants interpret these concepts consistently.

This concern is particularly important because participants were explicitly instructed regarding distinctions between pain and unpleasantness, and these explanations may themselves shape participant responses.

Accordingly:

i. the observed differentiation may partly reflect instructional framing;

ii. semantic interpretation may vary substantially across participants;

iii. and the findings may not necessarily represent stable underlying perceptual constructs.

The manuscript should acknowledge these construct-validity limitations more explicitly and avoid implying that these perceptual categories are objectively discrete entities.

3. VPTol methodology is fundamentally different from VPDT and VPPT

A major methodological issue is that VPTol was assessed using a substantially different protocol from VPDT and VPPT.

Specifically:

a. VPDT and VPPT used automated staircase procedures with reversal averaging;

b. VPTol used manually increased illumination over approximately 30 seconds;

c. VPTol was operator-controlled rather than algorithm-controlled;

d. VPTol was assessed only once per participant.

Consequently, VPTol may not represent a directly comparable psychophysical construct to VPDT and VPPT.

This introduces several important confounds, including:

i. temporal summation,

ii. adaptation effects,

iii. hysteresis effects,

iv. operator variability,

v. sequential conditioning,

vi. expectancy effects,

vii. and inconsistent stimulus kinetics.

Because of these methodological differences, direct quantitative comparison between VPTol and the other perceptual thresholds should be interpreted considerably more cautiously.

The manuscript acknowledges some of these issues in the Discussion; however, their implications are more substantial than currently presented.

At best, the authors should:

• explicitly discuss the non-equivalence of these paradigms;

• avoid implying direct comparability across all three measures;

• and clarify that VPTol remains exploratory.

4. Sequential testing may have conditioned participant responses

All participants underwent testing in the same sequence:

a. VPDT

b. VPPT

c. VPTol

This fixed ordering introduces potential:

i. anticipation effects,

ii. habituation,

iii. sensitisation,

iv. perceptual anchoring,

v. learning effects,

vi. and carryover conditioning.

Participants were repeatedly exposed to escalating light stimuli while simultaneously being instructed regarding the expected perceptual categories. Consequently, later responses may have been influenced by prior exposure and expectation rather than reflecting fully independent perceptual phenomena.

This issue is particularly relevant in psychophysical studies where repeated exposure may alter threshold perception over time.

Although the rationale for the testing sequence is understandable, the lack of counterbalancing should be discussed more explicitly as an important limitation.

5. Lack of test-retest reliability assessment

The manuscript repeatedly discusses the OPA as a quantitative sensory testing instrument suitable for clinical and research application. However, the present study provides no formal test-retest reliability assessment.

For psychophysical tools, reliability is fundamental.

The study would be substantially strengthened by reporting:

a. intra-session reliability,

b. repeatability coefficients,

c. intraclass correlation coefficients,

d. or threshold variability metrics.

Without such information, it remains difficult to determine whether the observed differences reflect stable perceptual phenomena or measurement variability.

6. Small sample size limits interpretability

The study includes only 29 participants.

While this may be acceptable for an exploratory psychophysical feasibility study, several interpretations are presented with a level of certainty more appropriate for a larger validation cohort.

Specific concerns include: limited statistical power, unstable subgroup analyses, inability to meaningfully assess sex differences and uncertain generalisability.

The manuscript should frame itself more explicitly as a preliminary or pilot investigation.

7. The healthy volunteer population limits external validity

The study exclusively examined healthy young adults with minimal ocular pain and minimal photophobia symptoms.

Accordingly:

A. the findings cannot be extrapolated directly to clinical photophobia populations;

B. pathological photosensitivity mechanisms remain untested;

C. the proposed clinical implications remain speculative.

In addition, the highly controlled experimental environment which includes dark adaptation, repeated exposures, scripted instructions, and controlled LED stimulation, may not adequately reflect real-world photophobia experiences.

The manuscript should therefore avoid overstating translational or clinical applicability. This is important.

8. Statistical reporting requires major improvement. Several aspects of the statistical analysis require clarification or improvement.

a) One-tailed testing

The use of one-tailed paired t-tests requires stronger justification. Unless highly specific directional hypotheses were prespecified a priori, two-tailed testing would generally be more appropriate.

b) Assumptions of RM-ANOVA

The manuscript does not indicate whether:

i. sphericity assumptions were assessed;

ii. whether corrections (such as Greenhouse-Geisser adjustment)s were applied where necessary.

c) Effect sizes

The manuscript reports p-values extensively but provides limited effect size reporting outside ηp² for one analysis.

Additional effect size metrics would improve interpretability.

d) Multiple testing burden

A large number of post hoc comparisons were conducted in a relatively small cohort. While Holm correction was applied, the manuscript should acknowledge the increased risk of unstable estimates.

9. The distinction between “pain” and “unpleasantness” may not be psychometrically robust

Participants were explicitly instructed regarding distinctions between pain intensity and unpleasantness. However:

a. the distinction may not have been uniformly understood;

b. repeated explanations may have introduced demand characteristics;

c. and the instructional framing itself may have shaped participant responses.

Accordingly, conclusions regarding “affective skewing” should be interpreted cautiously.

The current wording occasionally implies stronger mechanistic interpretation than the data justify.

10. The terminology proposal section is excessively speculative. Section 4.3 which advocates “photodysesthesia” and “photoallodynia” extends well beyond the actual empirical findings of the study.

While the conceptual discussion is intellectually interesting, the present dataset does not directly validate these proposed diagnostic or phenomenological constructs.

This section currently reads more as a conceptual position paper than an evidence-based interpretation of the experimental results. The authors should substantially shorten and temper this section. The proposed terminology framework is not empirically validated by the present study and appears disproportionate to the scope of the findings.

11. Potential conflict-of-interest implications should be handled with greater balance

Four of the authors are co-inventors of the OPA technology and may receive royalties from commercialisation.

The disclosure itself is appropriate and appreciated. However, portions of the Discussion and “Implications and future directions” section occasionally read more as advocacy for the device than objective evaluation.

The manuscript should ensure that:

i. claims regarding clinical applicability remain conservative;

ii. limitations are discussed with equal emphasis;

ii. the tone remains appropriately balanced.

MINOR COMMENTS

1. Clarify participant ocular status

Although major ocular disease was excluded, the manuscript does not clearly state whether participants underwent:

a. visual acuity testing,

b. slit-lamp examination,

c. refractive assessment,

d. tear-film evaluation,

e. formal ocular surface examination.

This should be clarified.

2. Clarify testing conditions more explicitly

The Methods section should specify:

a. whether testing was monocular or binocular;

b. ambient lighting conditions;

c. fixation requirements;

d, whether pupil size was monitored or controlled.

3. Improve terminology consistency

The manuscript alternates between:

i. “pain threshold”,

ii. “light-induced pain”,

iii. “photosensitivity”,

iv. “discomfort threshold”,

v. “photophobia”.

Terminology should be standardised where possible.

4. Figure clarity

Figures 3 and 4 are somewhat difficult to interpret visually in their current form. Improved labelling, clearer legends, and more transparent depiction of distributional characteristics would improve readability.

5. Reporting of VAS data

The manuscript alternates between means ± SD and medians with IQRs. While statistically justified, figure presentation should more clearly reflect the underlying distribution type.

6. Language and style

Several sentences in the Discussion are unnecessarily long and occasionally repetitive. Some claims could be rewritten more concisely and neutrally.

Examples include:

“marks an initial step towards…”

“substantially improve clinical diagnosis…”

“advance in our understanding…”

These could be simplified to improve precision and reduce interpretive overreach.

CONCLUSION

This manuscript presents an interesting and potentially useful psychophysical application of the Ocular Photosensitivity Analyzer for differentiating discomfort, pain, and tolerance responses to light in healthy adults.

The study appears ethically conducted and technically competent overall. However, several important methodological, psychophysical, and interpretive concerns currently limit confidence in the broader conclusions, particularly regarding:

construct validity, comparability of perceptual paradigms, psychometric robustness, reliability, generalisability, and clinical applicability.

Finally, the manuscript would benefit substantially from:

i. clearer methodological justification,

ii. stronger acknowledgment of psychophysical limitations,

iii. improved statistical reporting,

iv. more conservative interpretation of the findings.

With substantial revision, the manuscript may become suitable for publication in PLOS ONE.

**********

what does this mean?). If published, this will include your full peer review and any attached files.

If you choose “no”, your identity will remain anonymous but your review may still be made public.

**Do you want your identity to be public for this peer review?**  For information about this choice, including consent withdrawal, please see our Privacy Policy

Reviewer #1: No

Reviewer #2: No

**********

[NOTE: If reviewer comments were submitted as an attachment file, they will be attached to this email and accessible via the submission site. Please log into your account, locate the manuscript record, and check for the action link "View Attachments". If this link does not appear, there are no attachment files.]

To ensure your figures meet our technical requirements, please review our figure guidelines: https://journals.plos.org/plosone/s/figures

You may also use PLOS’s free figure tool, NAAS, to help you prepare publication quality figures: https://journals.plos.org/plosone/s/figures#loc-tools-for-figure-preparation.

NAAS will assess whether your figures meet our technical requirements by comparing each figure against our figure specifications.

# Author comment: Author response to Decision Letter 1

**DOI:** 10.1371/journal.pone.0361637.r002

7 Jul 2026

We have included a point-by-point response to reviewers in the "Response to Reviewers" document.

Attachment Submitted filename: Response to Reviewers.docx

# Peer review: Decision Letter 1

**DOI:** 10.1371/journal.pone.0361637.r003

**Authors:** Kofi Asiedu

7 Aug 2026

Dear Dr. Moulton,

Thank you for submitting your manuscript to PLOS ONE. After careful consideration, we feel that it has merit but does not fully meet PLOS ONE’s publication criteria as it currently stands. Therefore, we invite you to submit a revised version of the manuscript that addresses the points raised during the review process.

Please submit your revised manuscript by Sep 20 2026 11:59PM. If you will need more time than this to complete your revisions, please reply to this message or contact the journal office at plosone@plos.org. When you're ready to submit your revision, log on to https://www.editorialmanager.com/pone/ and select the 'Submissions Needing Revision' folder to locate your manuscript file.

- A letter that responds to each point raised by the academic editor and reviewer(s). You should upload this letter as a separate file labeled 'Response to Reviewers'.
- A marked-up copy of your manuscript that highlights changes made to the original version. You should upload this as a separate file labeled 'Revised Manuscript with Track Changes'.
- An unmarked version of your revised paper without tracked changes. You should upload this as a separate file labeled 'Manuscript'.

If you would like to make changes to your financial disclosure, please include your updated statement in your cover letter. Guidelines for resubmitting your figure files are available below the reviewer comments at the end of this letter.

If applicable, we recommend that you deposit your laboratory protocols in protocols.io to enhance the reproducibility of your results. Protocols.io assigns your protocol its own identifier (DOI) so that it can be cited independently in the future. For instructions see: https://journals.plos.org/plosone/s/submission-guidelines#loc-laboratory-protocols. Additionally, PLOS ONE offers an option for publishing peer-reviewed Lab Protocol articles, which describe protocols hosted on protocols.io. Read more information on sharing protocols at https://plos.org/protocols?utm_medium=editorial-email&utm_source=authorletters&utm_campaign=protocols.

As the corresponding author, your ORCID iD is verified in the submission system and will appear in the published article. PLOS supports the use of ORCID, and we encourage all coauthors to register for an ORCID iD and use it as well. Please encourage your coauthors to verify their ORCID iD within the submission system before final acceptance, as unverified ORCID iDs will not appear in the published article. *Only*  the individual author can complete the verification step; PLOS staff *cannot*  verify ORCID iDs on behalf of authors.

We look forward to receiving your revised manuscript.

Kind regards,

Kofi Asiedu, O.D., Ph.D. FAAO

Academic Editor

PLOS One

Journal Requirements:

If the reviewer comments include a recommendation to cite specific previously published works, please review and evaluate these publications to determine whether they are relevant and should be cited. There is no requirement to cite these works unless the editor has indicated otherwise.

[Note: HTML markup is below. Please do not edit.]

Reviewers' comments:

Reviewer's Responses to Questions

**Comments to the Author**

Reviewer #1: All comments have been addressed

Reviewer #2: All comments have been addressed

**********

2. Is the manuscript technically sound, and do the data support the conclusions?

Reviewer #1: Yes

Reviewer #2: Yes

**********

3. Has the statistical analysis been performed appropriately and rigorously?

Reviewer #1: Yes

Reviewer #2: Yes

**********

4. Have the authors made all data underlying the findings in their manuscript fully available?

The PLOS Data policy

Reviewer #1: Yes

Reviewer #2: Yes

**********

5. Is the manuscript presented in an intelligible fashion and written in standard English?

Reviewer #1: Yes

Reviewer #2: Yes

**********

Reviewer #1: Dear Authors,

Thank you for addressing the comments. I am satisfied with the revisions made and recommend the editor to accept the manuscript.

Reviewer #2: (No Response)

**********

what does this mean?). If published, this will include your full peer review and any attached files.

If you choose “no”, your identity will remain anonymous but your review may still be made public.

**Do you want your identity to be public for this peer review?**  For information about this choice, including consent withdrawal, please see our Privacy Policy

Reviewer #1: No

Reviewer #2: No

**********

[NOTE: If reviewer comments were submitted as an attachment file, they will be attached to this email and accessible via the submission site. Please log into your account, locate the manuscript record, and check for the action link "View Attachments". If this link does not appear, there are no attachment files.]

To ensure your figures meet our technical requirements, please review our figure guidelines: https://journals.plos.org/plosone/s/figures

You may also use PLOS’s free figure tool, NAAS, to help you prepare publication quality figures: https://journals.plos.org/plosone/s/figures#loc-tools-for-figure-preparation.

NAAS will assess whether your figures meet our technical requirements by comparing each figure against our figure specifications.

Attachment Submitted filename: Comments to Authors PLOS ONE OPS PAPER.docx

# Author comment: Author response to Decision Letter 2

**DOI:** 10.1371/journal.pone.0361637.r004

11 Aug 2026

We thank the reviewers for their time and thoughtful evaluation of our manuscript. As noted in their comments, no further revisions were requested. We provide formal responses below to comply with the journal’s resubmission requirements.

Reviewer 1

Comment: “All concerns have been addressed; the manuscript is recommended for publication.”

Response: We appreciate the reviewer’s careful assessment and positive recommendation. No additional revisions were requested, and no changes have been made to the manuscript.

Reviewer 2

Comment: “The authors have satisfactorily addressed all comments.”

Response: We thank the reviewer for their supportive evaluation. As no further modifications were requested, the manuscript remains unchanged.

Summary of Revisions

No revisions were required or made. A tracked changes version has been provided containing a brief note indicating that no changes were requested, to satisfy system requirements.

Attachment Submitted filename: Response to Reviewers-26.08.11.docx

# Editor report: Decision Letter 2

**DOI:** 10.1371/journal.pone.0361637.r005

**Authors:** Kofi Asiedu

14 Aug 2026

Assessment of light sensitivity using the Ocular Photosensitivity Analyzer: discomfort threshold, pain threshold, and tolerance in healthy volunteers

PLOS One

Dear Dr. Moulton,

Thank you for submitting your manuscript to PLOS ONE. After careful consideration, we feel that it has merit but does not fully meet PLOS ONE’s publication criteria as it currently stands. Therefore, we invite you to submit a revised version of the manuscript that addresses the points raised during the review process.

Kindly see the attached reviewer 2 comments. The requested revisions were not addressed. Incase authors were unable to see the review comments, they should contact the editorial office,

Please submit your revised manuscript by Sep 27 2026 11:59PM. If you will need more time than this to complete your revisions, please reply to this message or contact the journal office at plosone@plos.org. When you're ready to submit your revision, log on to https://www.editorialmanager.com/pone/ and select the 'Submissions Needing Revision' folder to locate your manuscript file.

Please include the following items when submitting your revised manuscript:

- A letter that responds to each point raised by the academic editor and reviewer(s). You should upload this letter as a separate file labeled 'Response to Reviewers'.
- A marked-up copy of your manuscript that highlights changes made to the original version. You should upload this as a separate file labeled 'Revised Manuscript with Track Changes'.
- An unmarked version of your revised paper without tracked changes. You should upload this as a separate file labeled 'Manuscript'.

If you would like to make changes to your financial disclosure, please include your updated statement in your cover letter. Guidelines for resubmitting your figure files are available below the reviewer comments at the end of this letter.

If applicable, we recommend that you deposit your laboratory protocols in protocols.io to enhance the reproducibility of your results. Protocols.io assigns your protocol its own identifier (DOI) so that it can be cited independently in the future. For instructions see: https://journals.plos.org/plosone/s/submission-guidelines#loc-laboratory-protocols. Additionally, PLOS ONE offers an option for publishing peer-reviewed Lab Protocol articles, which describe protocols hosted on protocols.io. Read more information on sharing protocols at https://plos.org/protocols?utm_medium=editorial-email&utm_source=authorletters&utm_campaign=protocols.

As the corresponding author, your ORCID iD is verified in the submission system and will appear in the published article. PLOS supports the use of ORCID, and we encourage all coauthors to register for an ORCID iD and use it as well. Please encourage your coauthors to verify their ORCID iD within the submission system before final acceptance, as unverified ORCID iDs will not appear in the published article. *Only*  the individual author can complete the verification step; PLOS staff *cannot*  verify ORCID iDs on behalf of authors.

We look forward to receiving your revised manuscript.

Kind regards,

Kofi Asiedu, O.D., Ph.D. FAAO

Academic Editor

PLOS One

Note from the Journal Office: Please find the comments attached as a seperate document. In case you cannot open the document, the reviewer comments have been added below:

**Comments to the authors**

This manuscript evaluates discomfort threshold, pain threshold, and tolerance to light using the Ocular Photosensitivity Analyzer in 29 healthy young adults. The topic is relevant, and the study may provide useful exploratory evidence that participants identify a higher illuminance as painful than as uncomfortable. However, several methodological and reporting issues currently prevent the central conclusions from being fully supported.

**Major comments**

**1. The three outcomes were not measured using equivalent procedures.**

VPDT and VPPT were assessed with an automated staircase and calculated from ten response reversals. VPTol was measured once using an operator-controlled gradual ramp. Therefore, the VPPT–VPTol difference is confounded by stimulus delivery, duration, operator involvement, and measurement reliability. VPTol should be treated as a separate exploratory outcome rather than as an equivalent third level in a validated three-level continuum. The abstract, Results, Discussion, and Conclusions should be revised accordingly.

**2. Perceptual level is confounded with test order.**

All participants completed VPDT, VPPT, and VPTol in that fixed sequence. Consequently, the observed ordering may partly reflect anchoring, expectancy, learning, sensitization, or habituation. The study cannot distinguish these order effects from differences among the constructs. This should be made explicit, and statements that the study demonstrates three distinct sensory experiences should be substantially moderated.

**3. A Discussion statement contradicts the statistical results.**

The Results state that VPTol pain intensity was not significantly greater than VPDT pain intensity (p=0.065) and that VPTol did not differ significantly from VPPT in either pain intensity or unpleasantness. The Discussion nevertheless states that both unpleasantness and pain intensity were significantly greater for VPTol and VPPT than for VPDT. Please correct this statement and verify all corresponding claims in the abstract, figure legends, and conclusion.

**4. Statistical reporting and missing-data handling require clarification.**

Please report the exact sample size included in every omnibus and pairwise analysis, explain the varying degrees of freedom, and state how missing observations were handled. The reported Mauchly result is incomplete—“Mauchly’s W=0.82, p )”—and should be corrected. Please also ensure that Holm-adjusted p-values are identified consistently and that confidence intervals are provided for the primary differences where feasible.

**5. The interpretation should remain exploratory.**

The study did not establish construct validity, test–retest reliability, clinical diagnostic performance, or abnormal reference ranges. The data show that participants selected different light levels in response to different instructions, with VPTol additionally assessed using a different procedure. They do not yet validate discomfort, pain, and tolerance as three distinct and valid OPA constructs. The proposed terms “photodysesthesia” and “photoallodynia” may remain as hypotheses for future research, but they should not be presented as empirically validated by this dataset.

**Minor comments**

1. Report non-normally distributed VAS outcomes consistently as medians and IQRs. The current text mixes median/IQR reporting with values formatted as mean ± SD.
2. Describe participants as individuals without relevant self-reported conditions, because no formal ocular examinations were performed.
3. Provide sufficient technical information for replication, including the photometer manufacturer and model, calibration approach, stimulus geometry, and the procedure and approximate rate used for the manual VPTol ramp.
4. Clarify whether the clear lenses were plano lenses and whether lens material, coating, and other relevant properties were standardized across participants.
5. State whether any participant reached the device’s maximum output before reporting pain or intolerance and explain how such observations were handled analytically.
6. Explain how the instruction to indicate intolerance “without squinting” was operationalized, how squinting was monitored, and what occurred if a participant squinted involuntarily.
7. Provide de-identified participant-level data and, preferably, analysis syntax. Figures should show the paired individual observations rather than only aggregate bars or box plots.
8. Correct the following textual issues: “VPTol exceed VPPT” should be “VPTol exceeded VPPT”; “differences from VPDT and VPDT testing” presumably should read “differences from VPDT and VPPT testing”; and “Helsinki Accord” should be replaced with “Declaration of Helsinki.”
9. Explain why assent is mentioned when all analyzed participants were aged 19–34 years.
10. Consider separating Results and Discussion. At minimum, distinguish direct findings clearly from interpretation.

**Recommendation: MAJOR REVISION**

The study has publishable exploratory potential, but its conclusions must be brought into closer alignment with the procedures and statistical findings. In particular, VPTol should be presented as an exploratory outcome, the implications of the fixed testing order should be clearly acknowledged, and the statistical and reporting inconsistencies should be corrected. The manuscript may become suitable for publication if the authors adequately address these issues and substantially moderate claims that cannot be supported by the present design.

Journal Requirements:

If the reviewer comments include a recommendation to cite specific previously published works, please review and evaluate these publications to determine whether they are relevant and should be cited. There is no requirement to cite these works unless the editor has indicated otherwise.

[Note: HTML markup is below. Please do not edit.]

[NOTE: If reviewer comments were submitted as an attachment file, they will be attached to this email and accessible via the submission site. Please log into your account, locate the manuscript record, and check for the action link "View Attachments". If this link does not appear, there are no attachment files.]

To ensure your figures meet our technical requirements, please review our figure guidelines: https://journals.plos.org/plosone/s/figures

You may also use PLOS’s free figure tool, NAAS, to help you prepare publication quality figures: https://journals.plos.org/plosone/s/figures#loc-tools-for-figure-preparation.

NAAS will assess whether your figures meet our technical requirements by comparing each figure against our figure specifications.

# Author comment: Author response to Decision Letter 3

**DOI:** 10.1371/journal.pone.0361637.r006

15 Sep 2026

We thank the reviewer for their comments and have revised the manuscript accordingly. Our responses are included in the "Response to reviewers" document.

Attachment Submitted filename: Response_to_reviewers_auresp_3.docx

# Peer review: Decision Letter 3

**DOI:** 10.1371/journal.pone.0361637.r007

**Authors:** Kofi Asiedu

14 Oct 2026

Assessment of light sensitivity using the Ocular Photosensitivity Analyzer: discomfort threshold, pain threshold, and tolerance in healthy volunteers

PONE-D-26-15230R3

Dear Dr. Moulton,

We’re pleased to inform you that your manuscript has been judged scientifically suitable for publication and will be formally accepted for publication once it meets all outstanding technical requirements.

Within one week, you’ll receive an e-mail detailing the required amendments. When these have been addressed, you’ll receive a formal acceptance letter and your manuscript will be scheduled for publication.

An invoice will be generated when your article is formally accepted. Please note, if your institution has a publishing partnership with PLOS and your article meets the relevant criteria, all or part of your publication costs will be covered. Please make sure your user information is up-to-date by logging into Editorial Manager at Editorial Manager® and clicking the ‘Update My Information' link at the top of the page. For questions related to billing, please contact billing support.

If your institution or institutions have a press office, please notify them about your upcoming paper to help maximize its impact. If they’ll be preparing press materials, please inform our press team as soon as possible -- no later than 48 hours after receiving the formal acceptance. Your manuscript will remain under strict press embargo until 2 pm Eastern Time on the date of publication. For more information, please contact onepress@plos.org.

Kind regards,

Kofi Asiedu, O.D., Ph.D. FAAO

Academic Editor

PLOS One

Additional Editor Comments (optional):

Reviewers' comments:

Reviewer's Responses to Questions

**Comments to the Author**

Reviewer #2: All comments have been addressed

**********

2. Is the manuscript technically sound, and do the data support the conclusions?

Reviewer #2: Yes

**********

3. Has the statistical analysis been performed appropriately and rigorously?

Reviewer #2: Yes

**********

4. Have the authors made all data underlying the findings in their manuscript fully available?

The PLOS Data policy

Reviewer #2: Yes

**********

5. Is the manuscript presented in an intelligible fashion and written in standard English?

Reviewer #2: Yes

**********

Reviewer #2: The authors have satisfactorily addressed all the queries and concerns raised during the review process. The revisions have substantially strengthened the scientific rigour and overall quality of the manuscript. I am satisfied with the revisions and have no further concerns.

**********

what does this mean?). If published, this will include your full peer review and any attached files.

If you choose “no”, your identity will remain anonymous but your review may still be made public.

**Do you want your identity to be public for this peer review?**  For information about this choice, including consent withdrawal, please see our Privacy Policy

Reviewer #2: No

**********

# Editor report: Acceptance letter

**DOI:** 10.1371/journal.pone.0361637.r008

**Authors:** Kofi Asiedu

PONE-D-26-15230R3

PLOS One

Dear Dr. Moulton,

I'm pleased to inform you that your manuscript has been deemed suitable for publication in PLOS One. Congratulations! Your manuscript is now being handed over to our production team.

At this stage, our production department will prepare your paper for publication. This includes ensuring the following:

* All references, tables, and figures are properly cited

* All relevant supporting information is included in the manuscript submission,

* There are no issues that prevent the paper from being properly typeset

You will receive further instructions from the production team, including instructions on how to review your proof when it is ready. Please keep in mind that we are working through a large volume of accepted articles, so please give us a few days to review your paper and let you know the next and final steps.

Lastly, if your institution or institutions have a press office, please let them know about your upcoming paper now to help maximize its impact. If they'll be preparing press materials, please inform our press team within the next 48 hours. Your manuscript will remain under strict press embargo until 2 pm Eastern Time on the date of publication. For more information, please contact onepress@plos.org.

You will receive an invoice from PLOS for your publication fee after your manuscript has reached the completed accept phase. If you receive an email requesting payment before acceptance or for any other service, this may be a phishing scheme. Learn how to identify phishing emails and protect your accounts at https://explore.plos.org/phishing.

If we can help with anything else, please email us at customercare@plos.org.

Thank you for submitting your work to PLOS One and supporting open access.

Kind regards,

PLOS One Editorial Office Staff

on behalf of

Dr. Kofi Asiedu

Academic Editor

PLOS One
